# Supplementary figures and images for: Role of the CTCF binding site in Human T-Cell Leukemia Virus-1 pathogenesis
Source: PLoS Pathog. 2025 Jun 3;21(6):e1012293. doi: 10.1371/journal.ppat.1012293 (PMC12165413; doi:10.1371/journal.ppat.1012293)

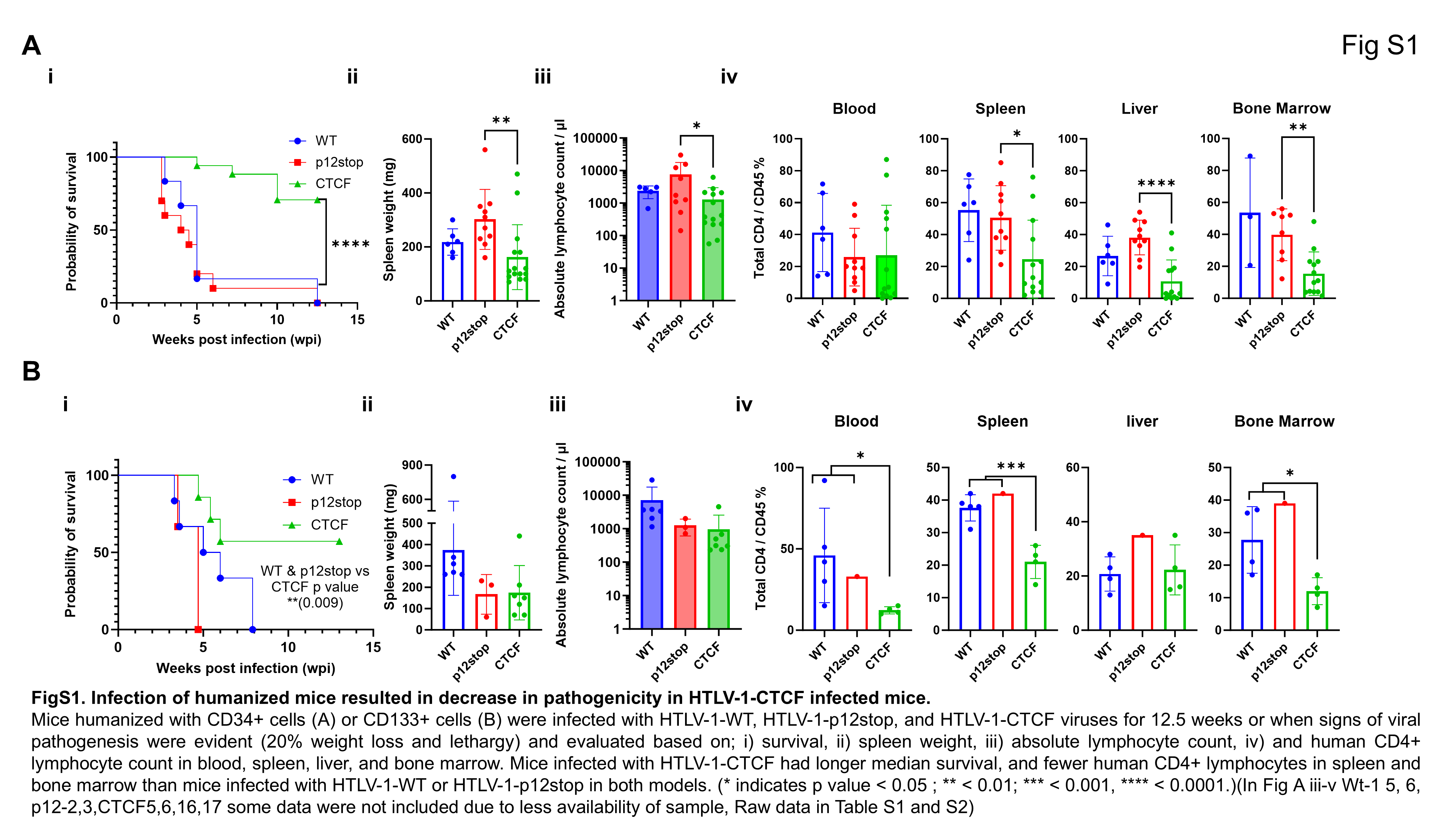

Supplement: S1 Fig — Mice humanized with CD34+ cells (A) or CD133+ cells (B) were infected with HTLV-1-WT, HTLV-1-p12stop, and HTLV-1-CTCF viruses for 12.5 weeks or when signs of viral pathogenesis were evident (20% weight loss and lethargy) and evaluated based on; i) survival, ii) spleen weight, iii) absolute lymphocyte count, iv) and human CD4+ lymphocyte count in blood, spleen, liver, and bone marrow. Mice infected with HTLV-1-CTCF had longer median survival, and fewer human CD4+ lymphocytes in spleen and bone marrow than mice infected with HTLV-1-WT or HTLV-1-p12stop in both models. (* indicates p value < 0.05; ** < 0.01; *** < 0.001, **** < 0.0001.)(In S1Aiii-S1Av Fig Wt-1 5, 6, p12-2,3,CTCF5,6,16,17 some data were not included due to less availability of sample, Raw data in S1 and S2 Tables). (PNG) [file ppat.1012293.s001.png]

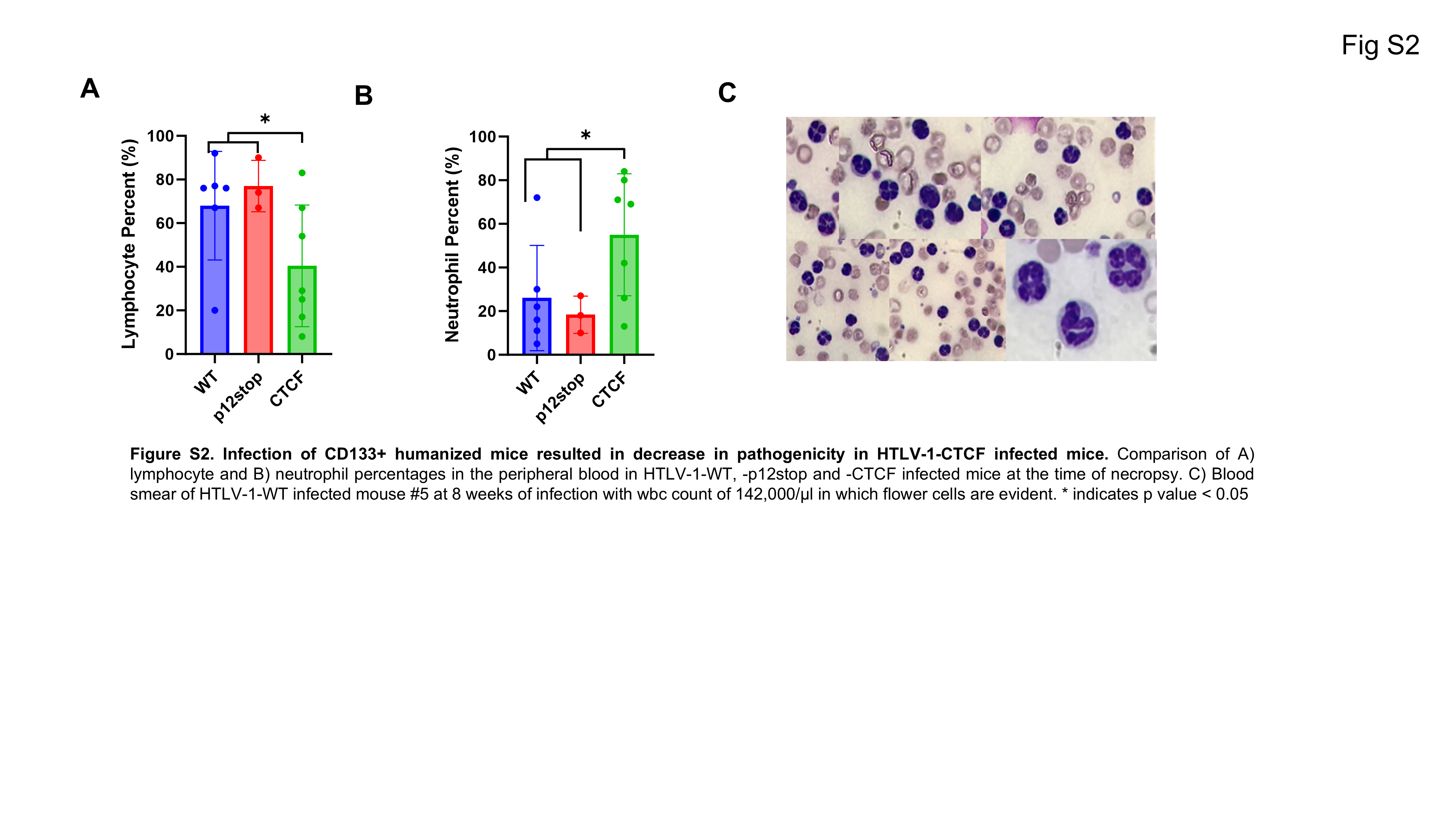

Supplement: S2 Fig — Comparison of A) lymphocyte and B) neutrophil percentages in the peripheral blood in HTLV-1-WT, -p12stop and -CTCF infected mice at the time of necropsy. C) Blood smear of HTLV-1-WT infected mouse #5 at 8 weeks of infection with wbc count of 142,000/µl in which flower cells are evident. * indicates p value < 0.05. (PNG) [file ppat.1012293.s002.png]

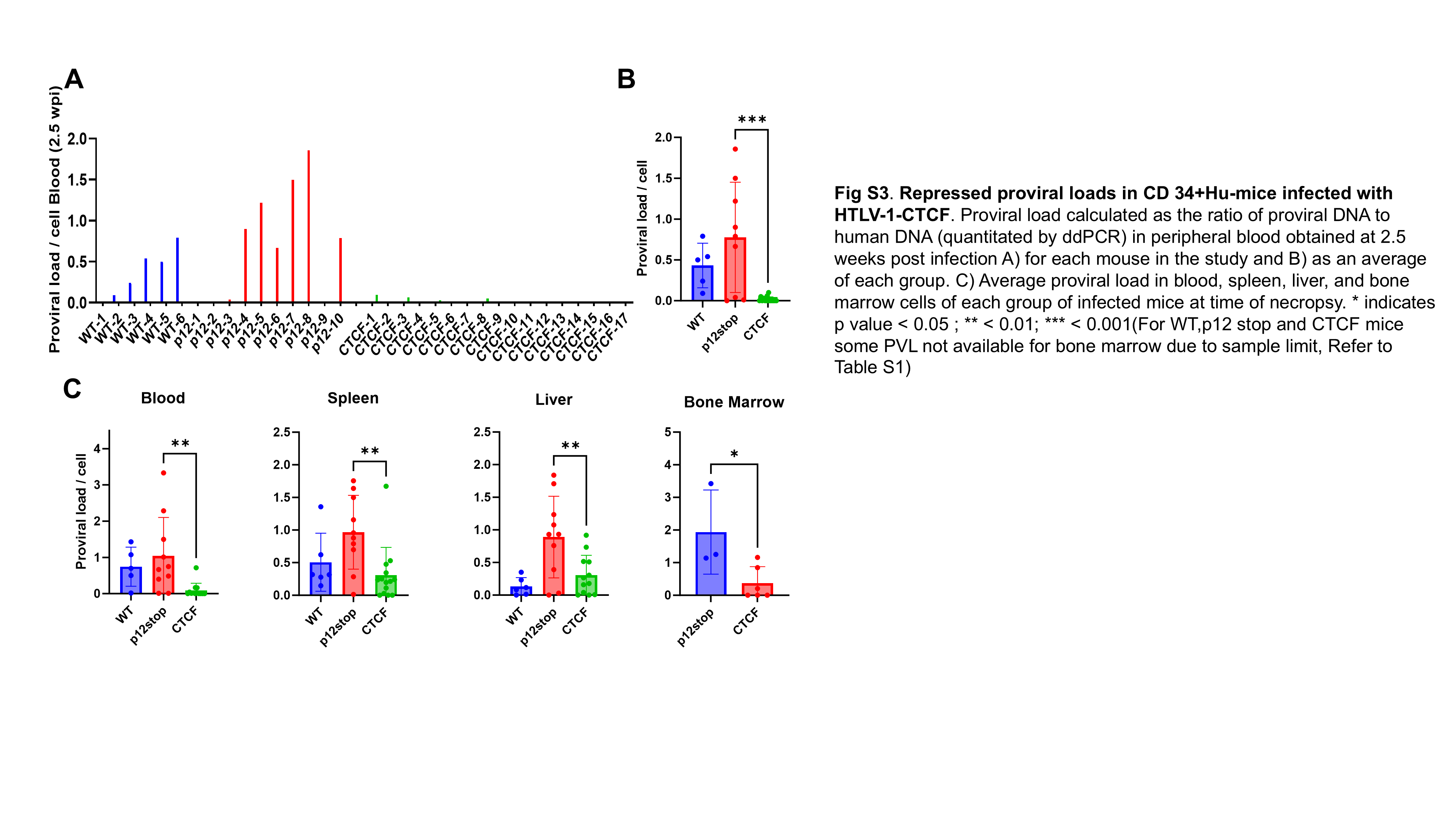

Supplement: S3 Fig — Proviral load calculated as the ratio of proviral DNA to human DNA (quantitated by ddPCR) in peripheral blood obtained at 2.5 weeks post infection A) for each mouse in the study and B) as an average of each group. C) Average proviral load in blood, spleen, liver, and bone marrow cells of each group of infected mice at time of necropsy. * indicates p value < 0.05; ** < 0.01; *** < 0.001(For WT,p12 stop and CTCF mice some PVL not available for bone marrow due to sample limit, Refer to S1 Table). (PNG) [file ppat.1012293.s003.png]

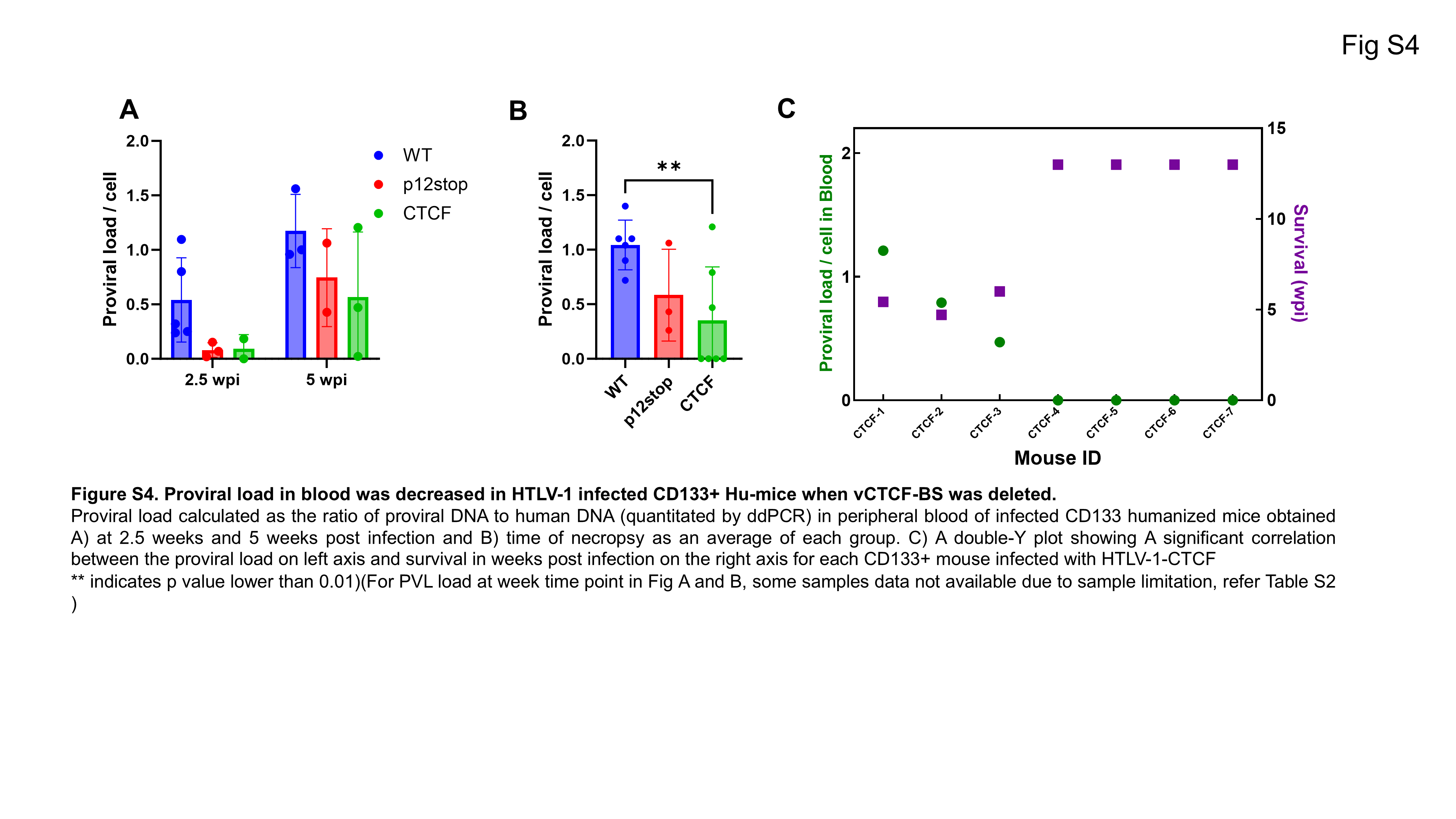

Supplement: S4 Fig — Proviral load calculated as the ratio of proviral DNA to human DNA (quantitated by ddPCR) in peripheral blood of infected CD133 humanized mice obtained A) at 2.5 weeks and 5 weeks post infection and B) time of necropsy as an average of each group. C) A double-Y plot showing A significant correlation between the proviral load on left axis and survival in weeks post infection on the right axis for each CD133+ mouse infected with HTLV-1-CTCF. ** indicates p value lower than 0.01)(For PVL load at week time point in S4A and S4B Figs, some samples data not available due to sample limitation, refer S2 Table) (PNG) [file ppat.1012293.s004.png]

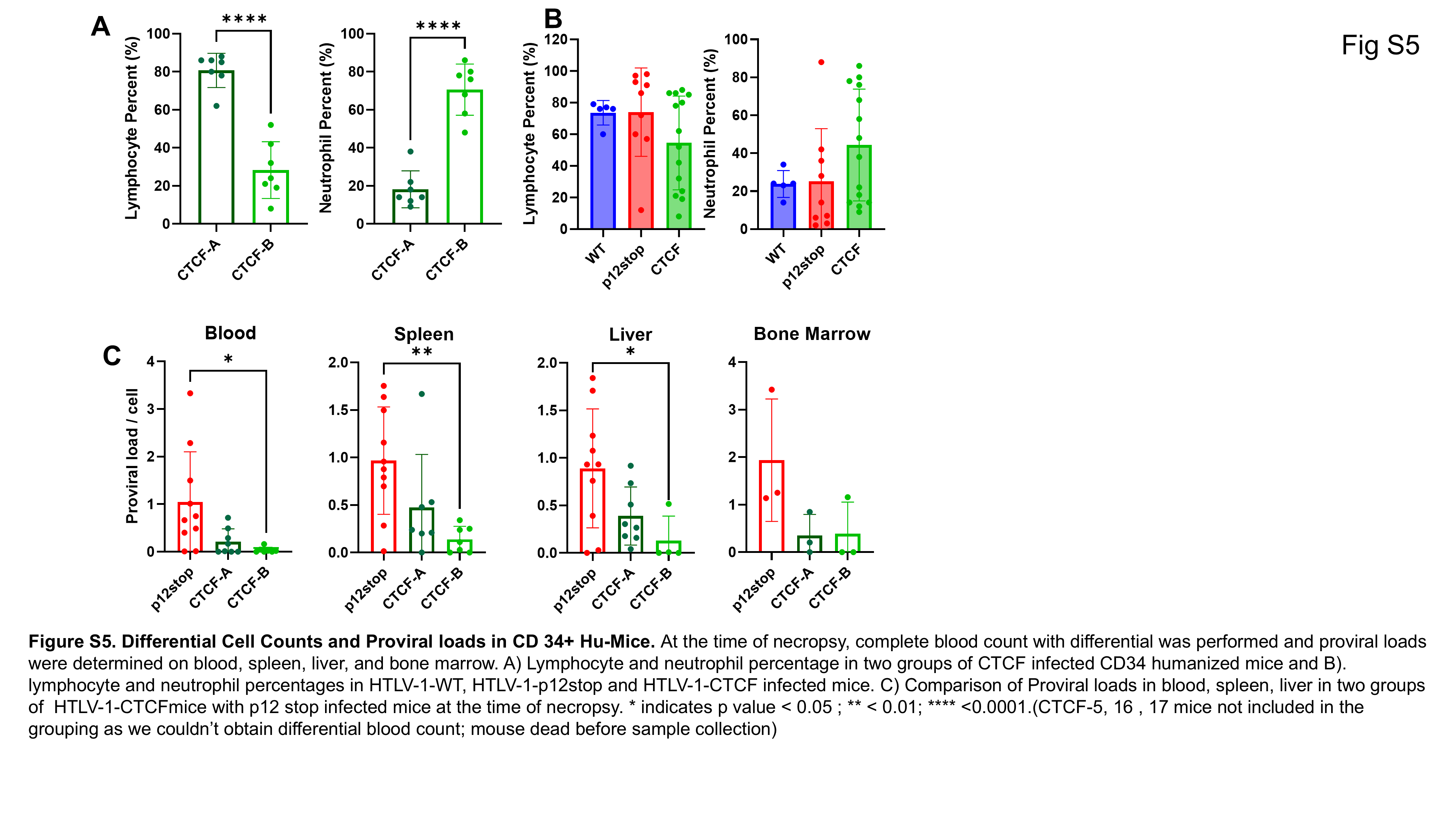

Supplement: S5 Fig — At the time of necropsy, complete blood count with differential was performed and proviral loads were determined on blood, spleen, liver, and bone marrow. A) Lymphocyte and neutrophil percentage in two groups of CTCF infected CD34 humanized mice and B). lymphocyte and neutrophil percentages in HTLV-1-WT, HTLV-1-p12stop and HTLV-1-CTCF infected mice. C) Comparison of Proviral loads in blood, spleen, liver in two groups of HTLV-1-CTCFmice with p12 stop infected mice at the time of necropsy. * indicates p value < 0.05; ** < 0.01; **** < 0.0001.(CTCF-5, 16, 17 mice not included in the grouping as we couldn’t obtain differential blood count; mouse dead before sample collection). (PNG) [file ppat.1012293.s005.png]

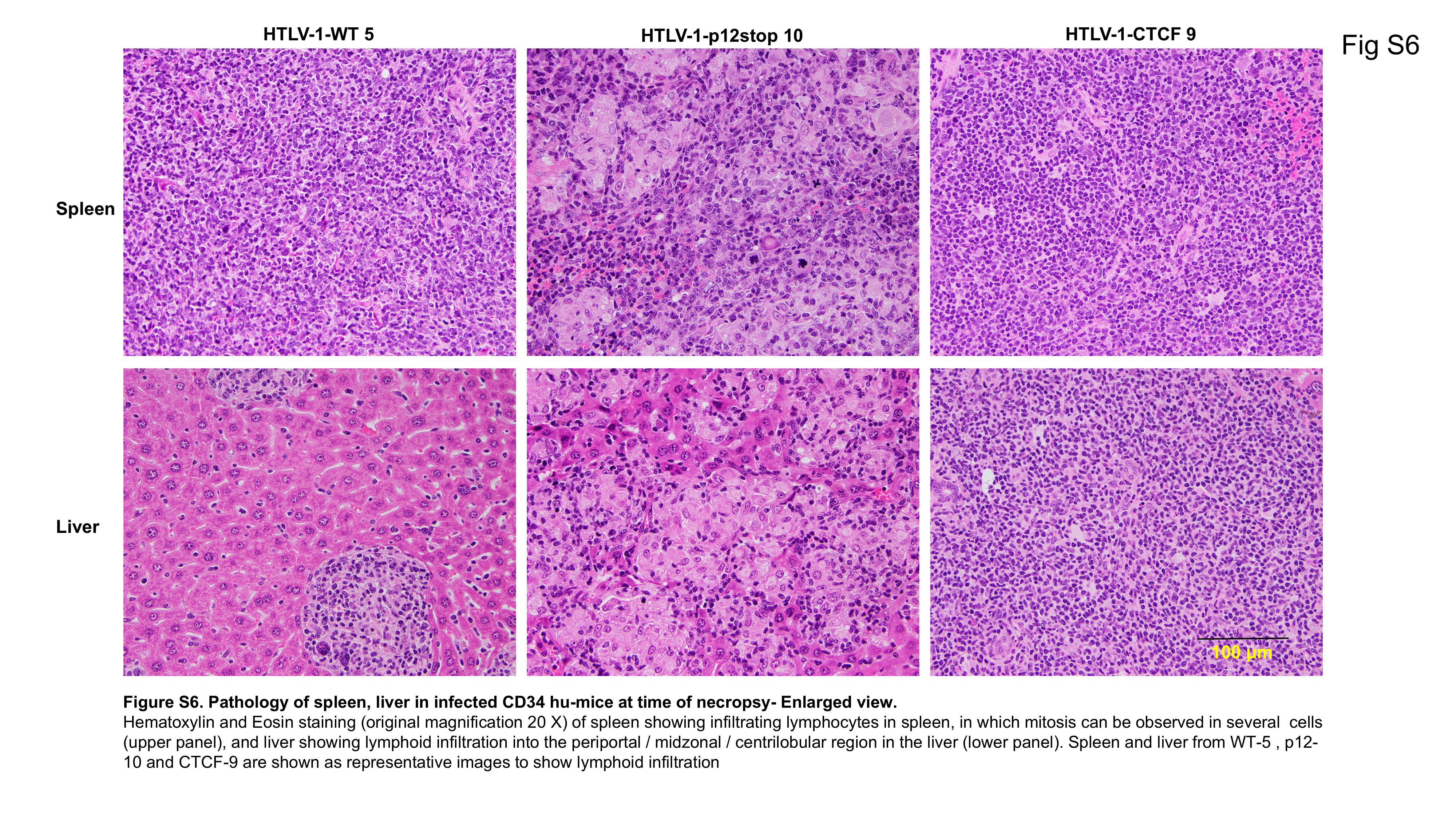

Supplement: S6 Fig — Hematoxylin and Eosin staining (original magnification 20 X) of spleen showing infiltrating lymphocytes in spleen, in which mitosis can be observed in several cells (upper panel), and liver showing lymphoid infiltration into the periportal/ midzonal/ centrilobular region in the liver (lower panel). Spleen and liver from WT-5, p12-10 and CTCF-9 are shown as representative images to show lymphoid infiltration. (PNG) [file ppat.1012293.s006.png]

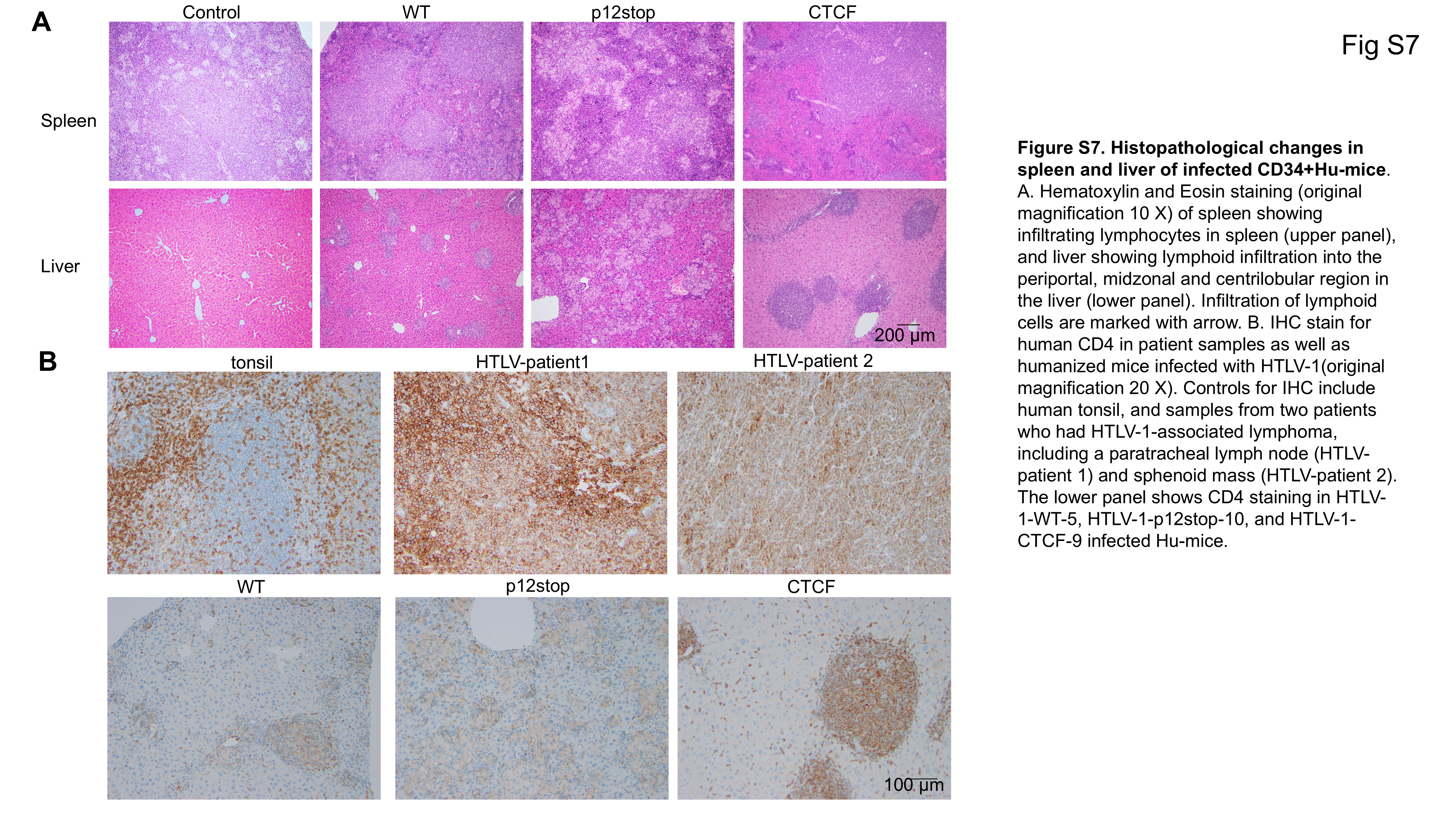

Supplement: S7 Fig — A. Hematoxylin and Eosin staining (original magnification 10 X) of spleen showing infiltrating lymphocytes in spleen (upper panel), and liver showing lymphoid infiltration into the periportal, midzonal and centrilobular region in the liver (lower panel). Infiltration of lymphoid cells are marked with arrow. B. IHC stain for human CD4 in patient samples as well as humanized mice infected with HTLV-1(original magnification 20 X). Controls for IHC include human tonsil, and samples from two patients who had HTLV-1-associated lymphoma, including a paratracheal lymph node (HTLV-patient 1) and sphenoid mass (HTLV-patient 2). The lower panel shows CD4 staining in HTLV-1-WT-5, HTLV-1-p12stop-10, and HTLV-1-CTCF-9 infected Hu-mice. (PNG) [file ppat.1012293.s007.png]

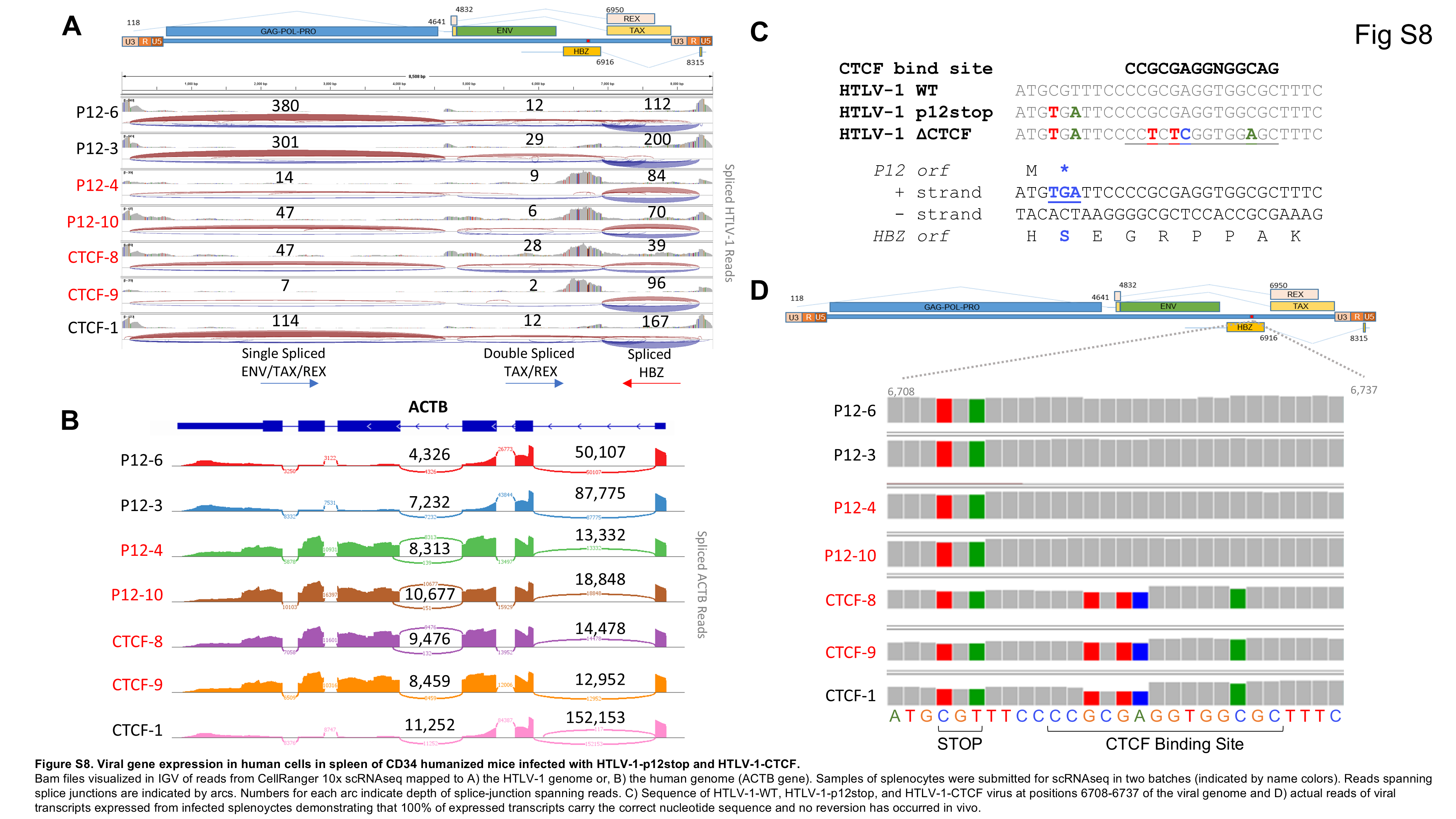

Supplement: S8 Fig — Bam files visualized in IGV of reads from CellRanger 10x scRNAseq mapped to A) the HTLV-1 genome or, B) the human genome (ACTB gene). Samples of splenocytes were submitted for scRNAseq in two batches (indicated by name colors). Reads spanning splice junctions are indicated by arcs. Numbers for each arc indicate depth of splice-junction spanning reads. C) Sequence of HTLV-1-WT, HTLV-1-p12stop, and HTLV-1-CTCF virus at positions 6708–6737 of the viral genome and D) actual reads of viral transcripts expressed from infected splenoyctes demonstrating that 100% of expressed transcripts carry the correct nucleotide sequence and no reversion has occurred in vivo. (PNG) [file ppat.1012293.s008.png]

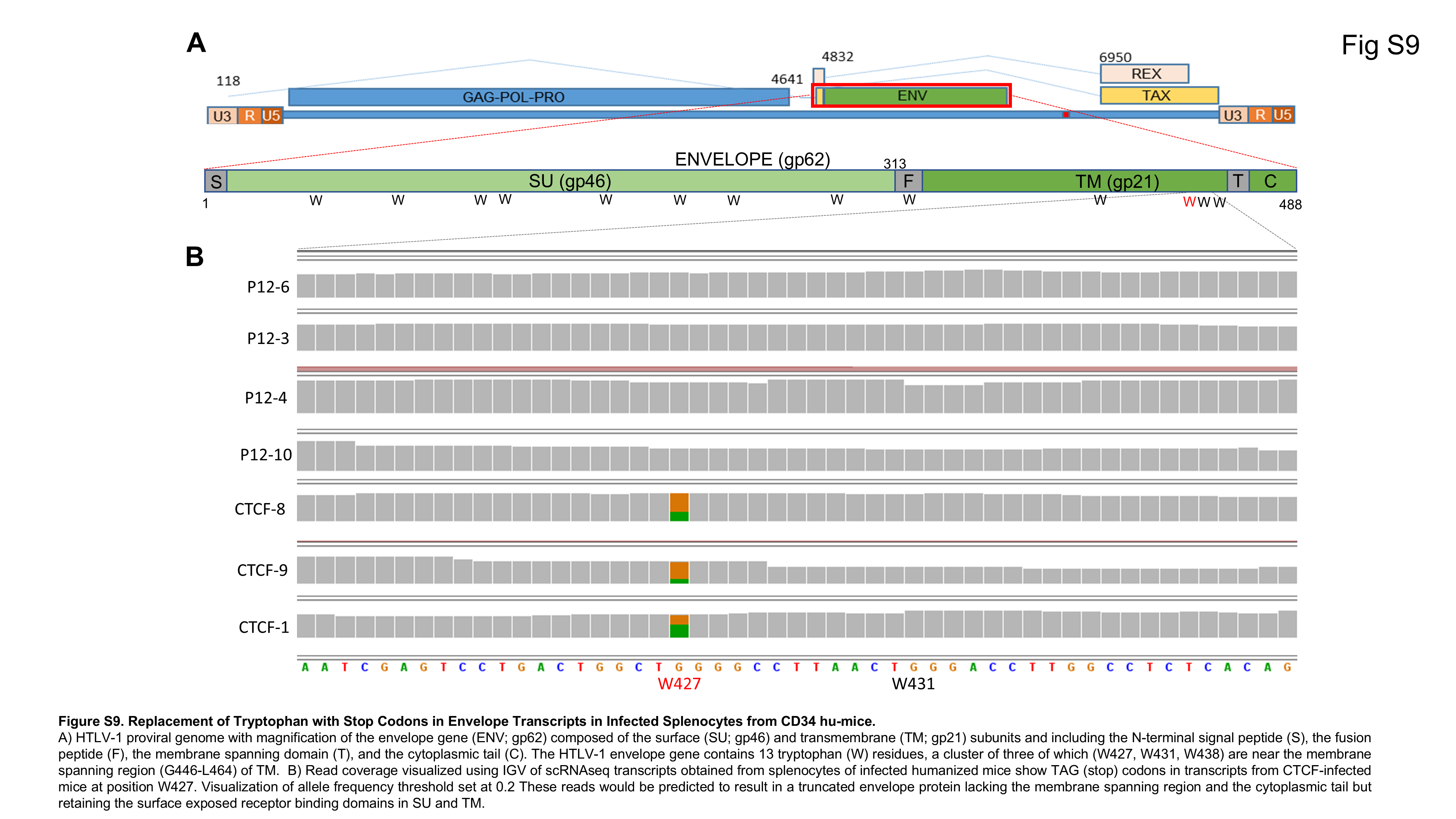

Supplement: S9 Fig — A) HTLV-1 proviral genome with magnification of the envelope gene (ENV; gp62) composed of the surface (SU; gp46) and transmembrane (TM; gp21) subunits and including the N-terminal signal peptide (S), the fusion peptide (F), the membrane spanning domain (T), and the cytoplasmic tail (C). The HTLV-1 envelope gene contains 13 tryptophan (W) residues, a cluster of three of which (W427, W431, W438) are near the membrane spanning region (G446-L464) of TM. B) Read coverage visualized using IGV of scRNAseq transcripts obtained from splenocytes of infected humanized mice show TAG (stop) codons in transcripts from CTCF-infected mice at position W427. Visualization of allele frequency threshold set at 0.2 These reads would be predicted to result in a truncated envelope protein lacking the membrane spanning region and the cytoplasmic tail but retaining the surface exposed receptor binding domains in SU and TM. (PNG) [file ppat.1012293.s009.png]

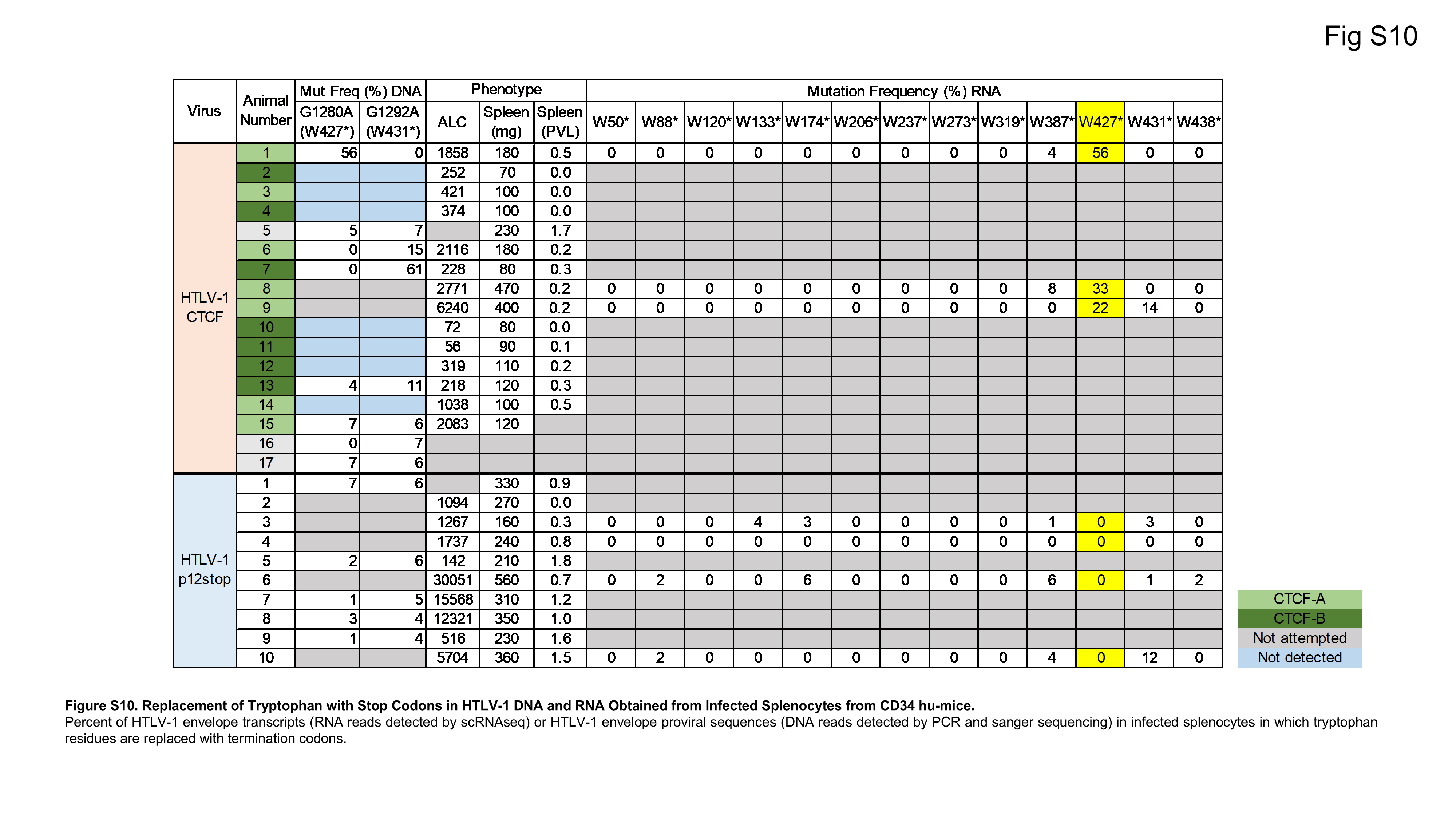

Supplement: S10 Fig — Percent of HTLV-1 envelope transcripts (RNA reads detected by scRNAseq) or HTLV-1 envelope proviral sequences (DNA reads detected by PCR and sanger sequencing) in infected splenocytes in which tryptophan residues are replaced with termination codons. (PNG) [file ppat.1012293.s010.png]

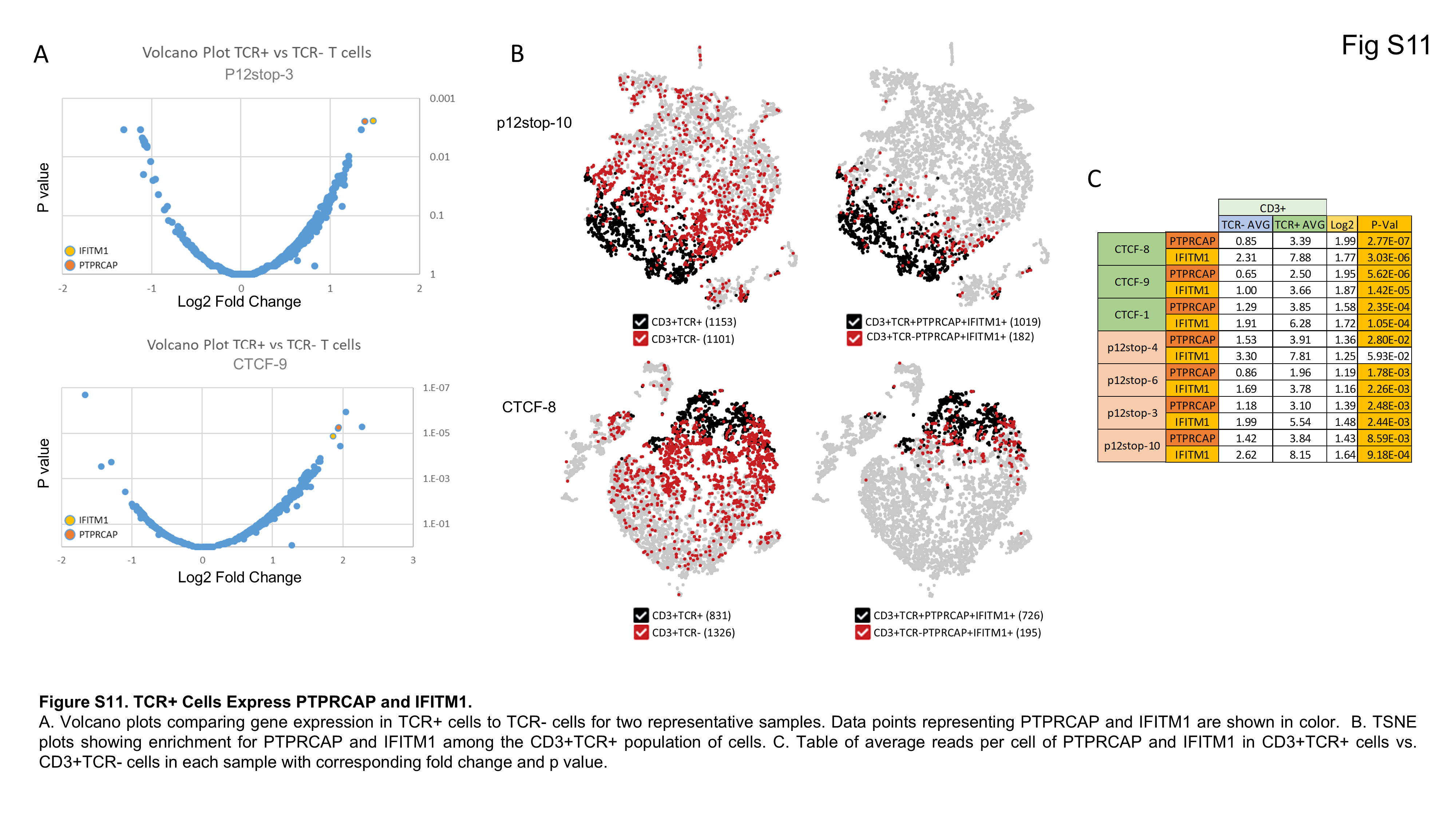

Supplement: S11 Fig — A. Volcano plots comparing gene expression in TCR+ cells to TCR- cells for two representative samples. Data points representing PTPRCAP and IFITM1 are shown in color. B. TSNE plots showing enrichment for PTPRCAP and IFITM1 among the CD3+ TCR+ population of cells. C. Table of average reads per cell of PTPRCAP and IFITM1 in CD3+ TCR+ cells vs. CD3+ TCR- cells in each sample with corresponding fold change and p value. (PNG) [file ppat.1012293.s011.png]

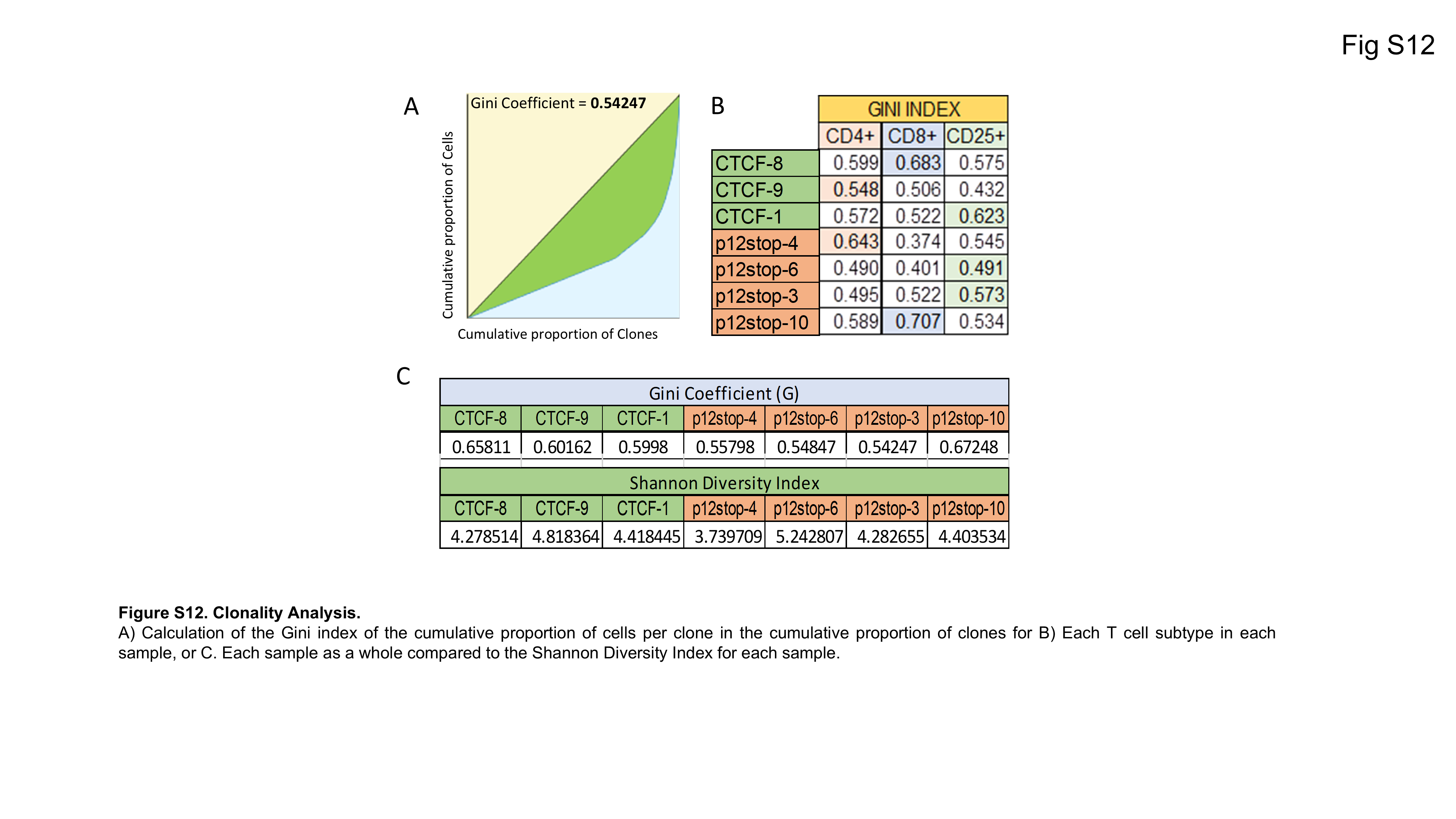

Supplement: S12 Fig — A) Calculation of the Gini index of the cumulative proportion of cells per clone in the cumulative proportion of clones for B) Each T cell subtype in each sample, or C. Each sample as a whole compared to the Shannon Diversity Index for each sample. (PNG) [file ppat.1012293.s012.png]

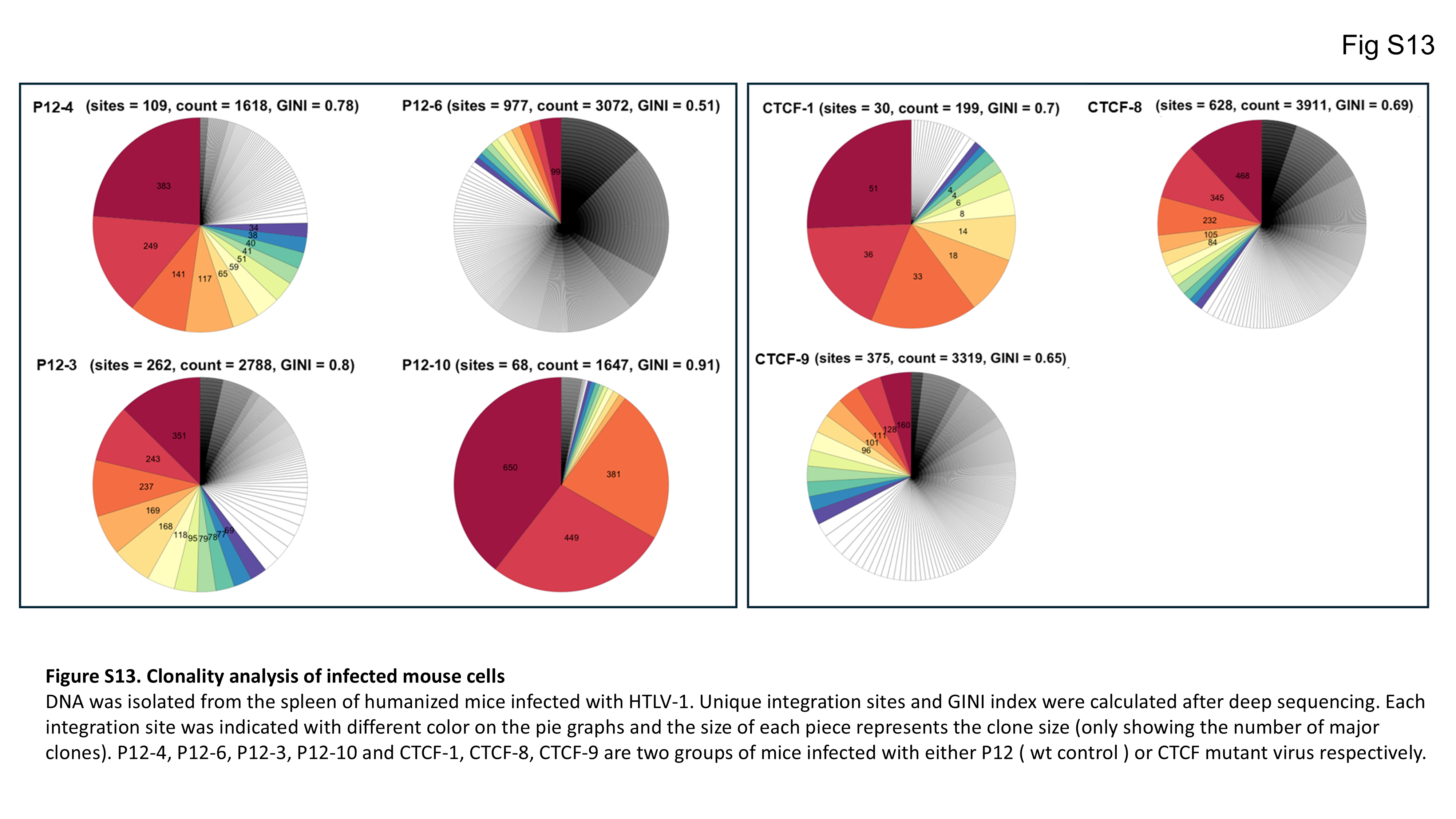

Supplement: S13 Fig — DNA was isolated from the spleen of humanized mice infected with HTLV-1. Unique integration sites and GINI index were calculated after deep sequencing. Each integration site was indicated with different color on the pie graphs and the size of each piece represents the clone size (only showing the number of major clones). P12-4, P12-6, P12-3, P12-10 and CTCF-1, CTCF-8, CTCF-9 are two groups of mice infected with either P12 (wt control) or CTCF mutant virus respectively. (PNG) [file ppat.1012293.s013.png]

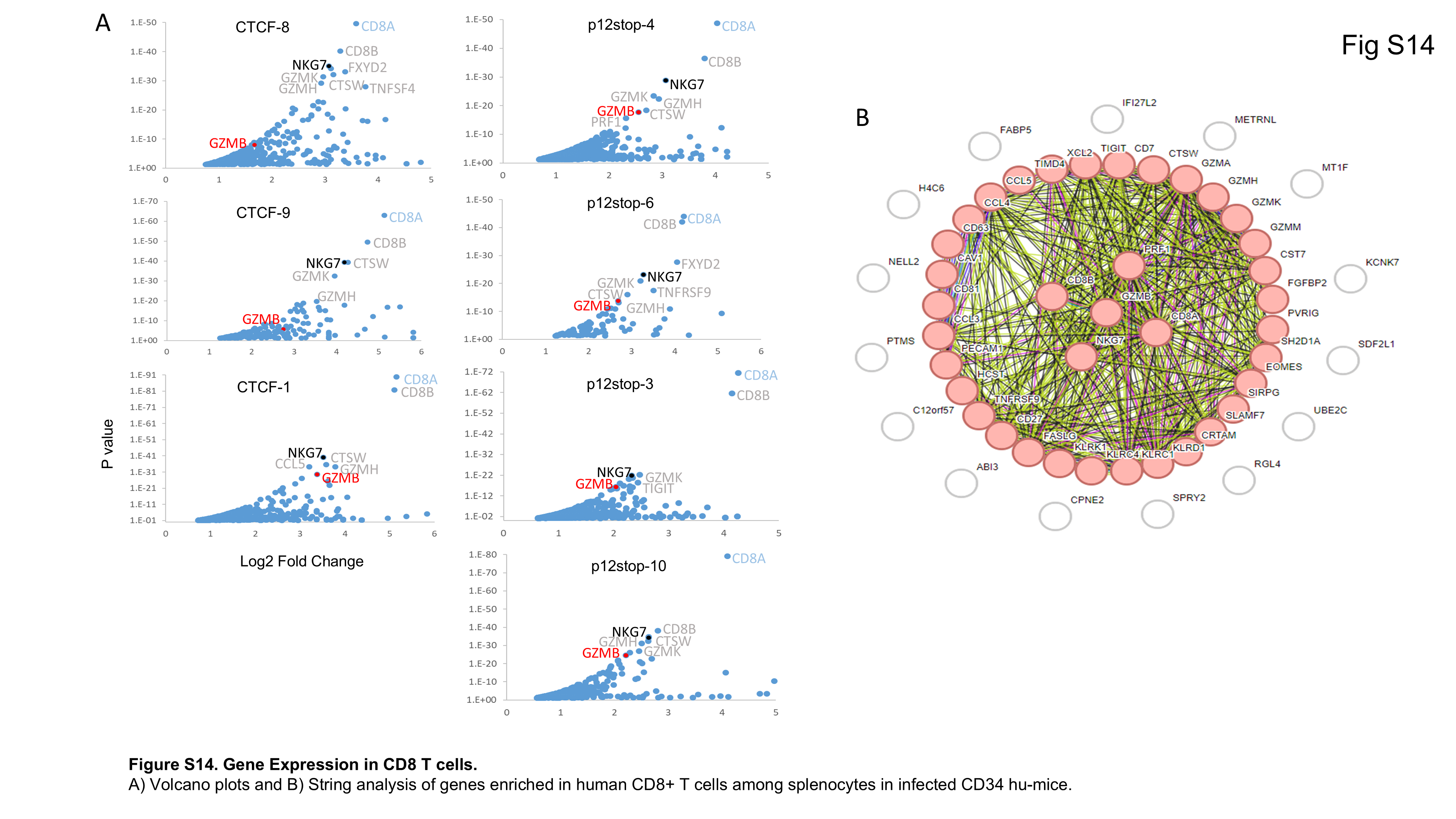

Supplement: S14 Fig — A) Volcano plots and B) String analysis of genes enriched in human CD8+ T cells among splenocytes in infected CD34 hu-mice. (PNG) [file ppat.1012293.s014.png]

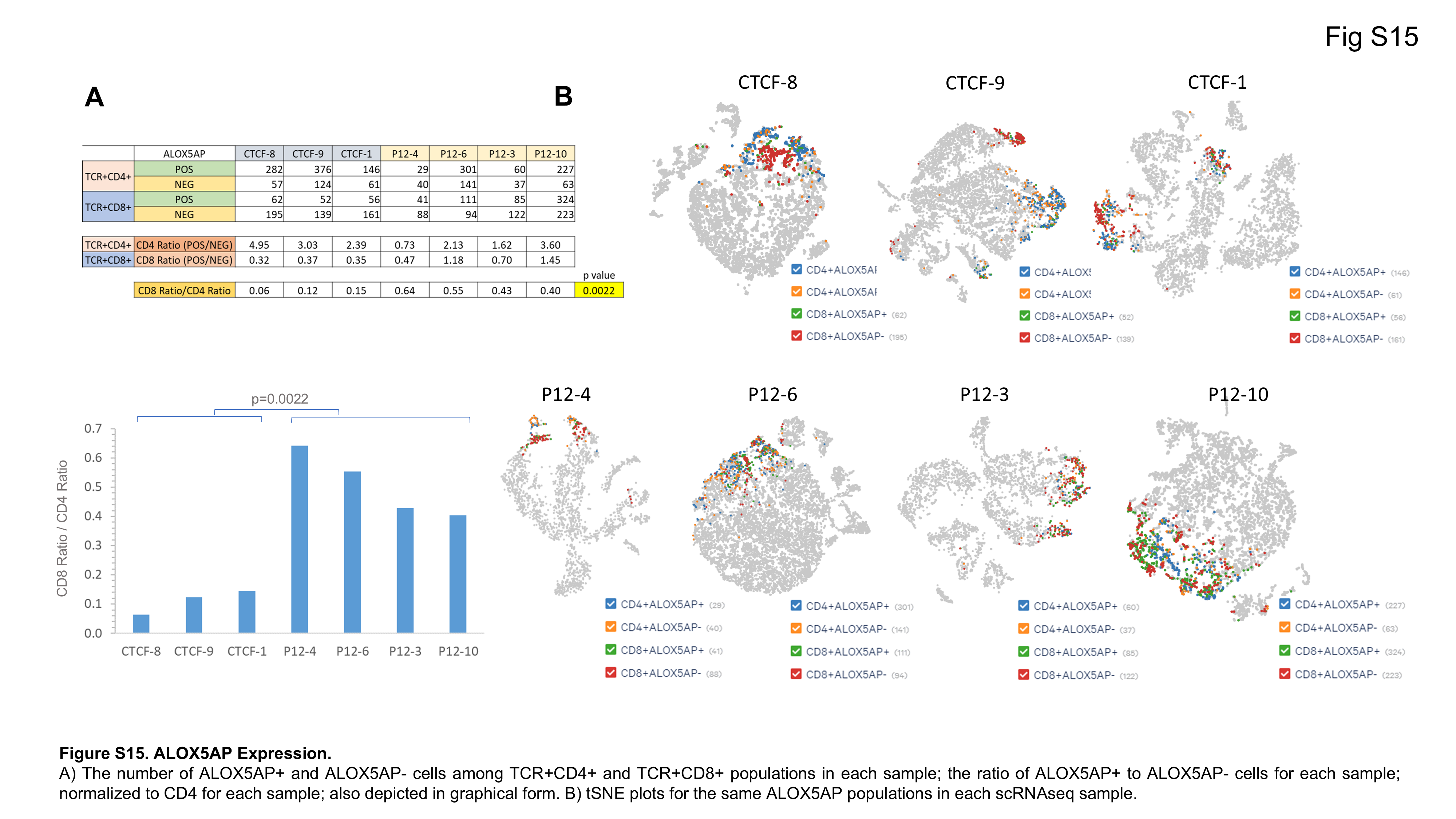

Supplement: S15 Fig — A) The number of ALOX5AP+ and ALOX5AP- cells among TCR+ CD4+ and TCR+ CD8+ populations in each sample; the ratio of ALOX5AP+ to ALOX5AP- cells for each sample; normalized to CD4 for each sample; also depicted in graphical form. B) tSNE plots for the same ALOX5AP populations in each scRNAseq sample. (PNG) [file ppat.1012293.s015.png]

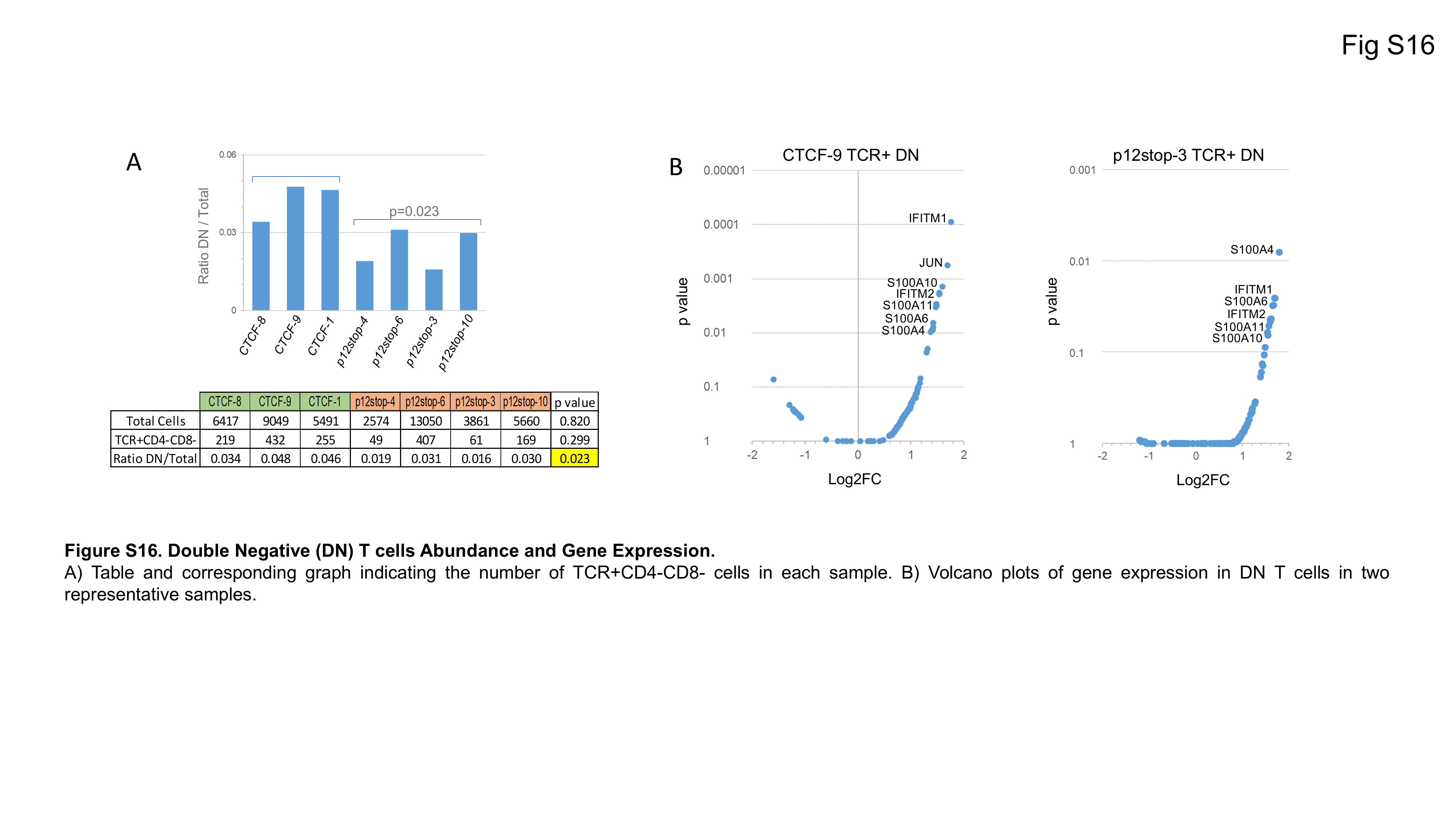

Supplement: S16 Fig — A) Table and corresponding graph indicating the number of TCR+ CD4-CD8- cells in each sample. B) Volcano plots of gene expression in DN T cells in two representative samples. (PNG) [file ppat.1012293.s016.png]

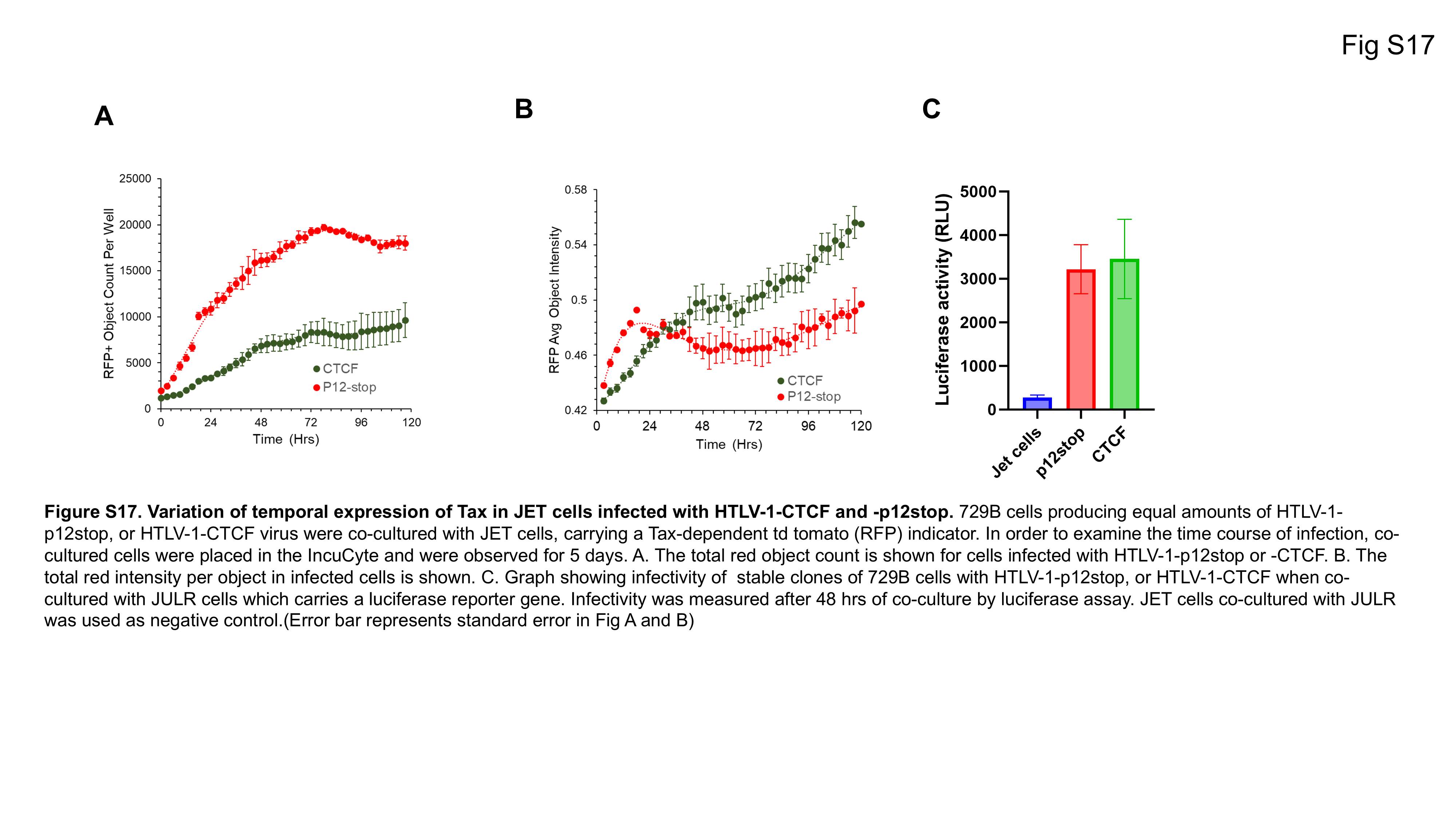

Supplement: S17 Fig — 729B cells producing equal amounts of HTLV-1-p12stop, or HTLV-1-CTCF virus were co-cultured with JET cells, carrying a Tax-dependent td tomato (RFP) indicator. In order to examine the time course of infection, co-cultured cells were placed in the IncuCyte and were observed for 5 days. A. The total red object count is shown for cells infected with HTLV-1-p12stop or -CTCF. B. The total red intensity per object in infected cells is shown. C. Graph showing infectivity of stable clones of 729B cells with HTLV-1-p12stop, or HTLV-1-CTCF when co-cultured with JULR cells which carries a luciferase reporter gene. Infectivity was measured after 48 hrs of co-culture by luciferase assay. JET cells co-cultured with JULR was used as negative control.(Error bar represents standard error in S17A and S17B Fig). (PNG) [file ppat.1012293.s017.png]
