# Supplementary material for: Role of the CTCF binding site in Human T-Cell Leukemia Virus-1 pathogenesis
Source: PLoS Pathog. 2025 Jun 3;21(6):e1012293. doi: 10.1371/journal.ppat.1012293 (PMC12165413; doi:10.1371/journal.ppat.1012293)
Supplement: S1 Table — (PDF) [file ppat.1012293.s018.pdf]

Table S1

Mouse new ID	Sacrifi (wpi)	FACS Data Total CD4/CD 45%				Blood at time of Necropsy			Abs Lymph count	spleen wt (mg)	PVL load at time of necropsy				Any Lymphnodes		Proviral load in blood /cell in every 2.5 Weeks post Infection			
		Blood	Spleen	Liver	BM	WBC count	% lymph	% neutro			Blood	spleen	Liver	BM						
**WT-1**	4	15%	70%	33%		3720	60%	34%	2232	240	0.017	0.317	0.351				2.5	5	7.5	10
**WT-2**	5	44%	24%	9%	51%	4040	76%	24%	3070	220	1.432	0.623	0.233				0.090	1.793		
**WT-3**	5	37%	60%	27%	89%	4060	76%	24%	3086	180	0.500	0.147	0.013				0.239	1.564		
**WT-4**	12.5	71.7%	77.5%	22.6%	20.6%	3600	77%	23%	2772	300	1.073	1.357	0.000				0.536	1.338	1.220	1.642
**WT-5**	3	66%	41%	46%						210		0.315	0.115				0.500			
**WT-6**	5	14%	59%	22%		860	79%	14%	675	160	0.698	0.278	0.083				0.791	1.347		
**p12-1**	12.5	4.9%	21.2%	19.6%	12.2%					330	3.330	0.877	1.234	3.423			0.000	0.000		0.000
**p12-2**	3	23%	38%	43%		1920	57%	42%	1094	270	0.009	0.014	0.000							
**p12-3**	4	10%	61%	36%		1760	72%	28%	1267	160	0.487	0.285	0.030				0.038			
**p12-4**	6	52%	65%	36%	52%	2020	86%	14%	1737	240	1.012	0.791	0.932	1.250			0.898			
**p12-5**	5	13%	29%	39%	29%	1180	12%	88%	142	210	2.286	1.755	1.840				1.220	2.286		
**p12-6**	4.5	19%	72%	49%	53%	30980	97%	3%	30051	560	0.666	0.698	0.391	1.139	0.57		0.666			
**p12-7**	2.8	39%	53%	33%	51%	16740	93%	7%	15568	310	1.500	1.157	1.707				1.500			
**p12-8**	2.8	59%	85%	48%	56%	13540	91%	6%	12321	350	0.400	0.957	0.759				1.857			
**p12-9**	2.8	20%	42%	24%	24%	860	60%	36%	516	230	0.010	1.638	1.076				0.010			
**p12-10**	5	19%	38%	54%	41%	5820	98%	2%	5704	360	0.747	1.498	0.930				0.789			
**CTCF-1**	12.5	87%	76%	41%	21%	2160	86%	14%	1858	180	0.000	0.476	0.735				0.097	0.138	0.239	0.105
**CTCF-2**	12.5	7%	4%	0%	4%	600	42%	58%	252	70	0.002	0.000					0.000	0.000	0.000	0.000
**CTCF-3**	12.5	50%	64%	4%	23%	540	78%	22%	421	100	0.011	0.000	0.040	0.000			0.064	0.009	0.050	0.123
**CTCF-4**	12.5	0%	10%	0%	23%	720	52%	48%	374	100	0.044	0.029		0.000			0.000	0.000	0.000	0.000
**CTCF-5**	10	Dead								230		1.672	0.917				0.029	0.200	0.490	
**CTCF-6**	7.2	54%				2460	86%	9%	2116	180	0.716	0.213	0.159				0.000	0.249		
**CTCF-7**	12.5	1.6%	9.3%	2.0%	4.4%	1200	19%	80%	228	80	0.163	0.251	0.515	1.160						
**CTCF-8**	12.5	43.4%	37.0%	17.6%	3.8%	3260	85%	14%	2771	470	0.000	0.237	0.178	0.205			0.052	0.080	0.000	0.007
**CTCF-9**	12.5	77.3%	48.9%	17.4%	9.9%	7800	80%	18%	6240	400	0.000	0.197	0.304	0.848			0.000	0.000	0.056	0.000
**CTCF-10**	12.5	0.4%	7.2%	0.1%	1.5%	300	24%	76%	72	80	0.000	0.000	0.000	0.000			NA	0.000		0.060
**CTCF-11**	12.5		2%	19%	4%	700	8%	86%	56	90	0.018	0.111	0.000				0.000	0.000	0.009	0.105
**CTCF-12**	12.5	3%	4%	1%	16%	1520	21%	78%	319	110	0.000	0.240	0.007				0.000	0.000	0.000	0.002
**CTCF-13**	12.5	6%	21%	3%	11%	680	32%	68%	218	120	0.021	0.344					0.000	0.000	0.000	0.000
**CTCF-14**	12.5	3%	23%	3%	31%	1180	88%	12%	1038	100	0.167	0.530	0.509				0.000	0.000	0.000	0.000
**CTCF-15**	5	18.0%	13%	31.0%	48.0%	3360	62%	38%	2083	120			0.266				0.000	0.289		
**CTCF-16**	10		Dead														0.000	0.000	1.490	
**CTCF-17**	10	52%	Dead														0.000	0.000	0.008	

Not analyzed due to insufficient sample

Mice deceased before the time point

CTCF-5 , 16 and 17 found dead
