# Supplementary material for: Role of the CTCF binding site in Human T-Cell Leukemia Virus-1 pathogenesis
Source: PLoS Pathog. 2025 Jun 3;21(6):e1012293. doi: 10.1371/journal.ppat.1012293 (PMC12165413; doi:10.1371/journal.ppat.1012293)
Supplement: S2 Table — (PDF) [file ppat.1012293.s019.pdf]

Table S2

	New mouse ID	Sacrifi (wpi)	FACS data CD4/cd45 %				CBC	DIFF		spleen wt(mg)	Abs Lymp count	Proviral load/cell in Blood at time of necropsy	PVL Weeks post Infection		
			Blood	Spleen	Liver	BM	WBC count	% lymph	% neutro				2.5	5	7.5
	WT-1	3.3	0.416	0.391	0.280	0.357	3640	0.92	0.05	260	3348.8	0.90	1.0959		
	WT-2	3.6	0.298	0.376	0.228	0.169	2680	0.76	0.11	270	2036.8	0.72	0.80		
	WT-3	6.0	0.277	0.365	0.128	0.135	1500	0.76	0.16	310	1140	1.10	0.24	1.56	
	WT-4	7.9	0.510	0.380	0.190	0.210	5440	0.67	0.30	260	3644.8	1.04	0.32	1.00	
	WT-5	7.9	0.920	0.420	0.130	0.370	142340	0.20	0.72	800	28468	1.10	0.25	0.96	
	WT-6	5.0	0.150	0.310			4900	0.77	0.22	340	3773	1.40			
	p12-1	4.70		0.157	0.053	0.536	1060	0.67	0.27	60	710.2	1.06	0.068489	1.062507	
	p12-2	3.50	0.327	0.422	0.351	0.391	2220	0.90	0.10	230	1998	0.26	0.0190		
	p12-3	4.70	0.002	0.159	0.132	0.192	4180	0.74	0.18	210	3093.2	0.43	0.152641	0.427518	
	CTCF-1	5.43	0.147	0.212	0.225	0.125	5440	0.83	0.13	150	4515.2	1.21	0.18452	1.206419	
	CTCF-2	4.71	0.095	0.227	0.349	0.074	740	0.67	0.26	160	495.8	0.79			
	CTCF-3	6.00	0.001	0.115	0.017	0.288	1260	0.54	0.42	440	680.4	0.47		0.47097	
	CTCF-4	13.00	0.108	0.139	0.153	0.111	1360	0.17	0.8	70	231.2	0			
	CTCF-5	13.00	0.127	0.259	0.160	0.172	3880	0.08	0.84	210	310.4	0	0	0.02266	0.0270
	CTCF-6	13.00					1040	0.29	0.69	70	301.6	0			
	CTCF-7	13.00					940	0.25	0.71	120	235	0			
P value	WT vs CTCF	0.046	0.025	0.000	0.870	0.161	0.271	0.089	0.073	0.060	0.156	0.009		0.200	
	P12 vs. CTCF	0.058	0.504	0.480	0.981	0.047	0.755	0.066	0.063	0.929	0.374	0.499		0.747	
	WT vs P12	0.327	0.267	0.077	0.873	0.248	0.497	0.580	0.620	0.159	0.443	0.064		0.302	
	WT+P12 vs CTCF	0.009	0.058	0.017	0.928	0.067	0.365	0.025	0.019	0.160	0.213	0.024		0.257	

Not analyzed due to insufficient sample

Mice deceased before the time point
