# Supplementary material for: Role of the CTCF binding site in Human T-Cell Leukemia Virus-1 pathogenesis
Source: PLoS Pathog. 2025 Jun 3;21(6):e1012293. doi: 10.1371/journal.ppat.1012293 (PMC12165413; doi:10.1371/journal.ppat.1012293)
Supplement: S3 Table — (PDF) [file ppat.1012293.s020.pdf]

Table S3

Groups	Mouse new ID	Sacrifi (wpi)	FACS Data Total CD4/CD 45%				Blood at time of Necropsy			Abs Lymh count	spleen wt (mg)	PVL load at time of necropsy				Any Lymphnodes		Proviral load in blood /cell in every 2.5			
			**Blood**	**Spleen**	**Liver**	**BM**	**WBC count**	**% lymph**	**% neutro**			**Blood**	**spleen**	**Liver**	**BM**			Weeks post Infection			
																		2.5	5	7.5	10
	**CTCF-1**	12.5	87%	76%	41%	21%	2160	86%	14%	1858	180	0.000	0.476	0.735				0.097	0.138	0.239	0.105
	**CTCF-6**	7.2	54%				2460	86%	9%	2116	180	0.716	0.213	0.159				0.000	0.249		
	**CTCF-8**	12.5	43.4%	37.0%	17.6%	3.8%	3260	85%	14%	2771	470	0.000	0.237	0.178	0.205			0.052	0.080	0.000	0.007
	**CTCF-9**	12.5	77.3%	48.9%	17.4%	9.9%	7800	80%	18%	6240	400	0.000	0.197	0.304	0.848			0.000	0.000	0.056	0.000
CTCF A	**CTCF-3**	12.5	50%	64%	4%	23%	540	78%	22%	421	100	0.011	0.000	0.040	0.000			0.064	0.009	0.050	0.123
	**CTCF-14**	12.5	3%	23%	3%	31%	1180	88%	12%	1038	100	0.167	0.530	0.509				0.000	0.000	0.000	0.000
	**CTCF-15**	5	18.0%	13%	31.0%	48.0%	3360	62%	38%	2083	120			0.266				0.000	0.289		
	Average		47%	43%	19%	23%															
	**CTCF-2**	12.5	7%	4%	0%	4%	600	42%	58%	252	70	0.002	0.000					0.000	0.000	0.000	0.000
	**CTCF-4**	12.5	0%	10%	0%	23%	720	52%	48%	374	100	0.044	0.029		0.000			0.000	0.000	0.000	0.000
	**CTCF-7**	12.5	1.6%	9.3%	2.0%	4.4%	1200	19%	80%	228	80	0.163	0.251	0.515	1.160						
CTCF B	**CTCF-10**	12.5	0.4%	7.2%	0.1%	1.5%	300	24%	76%	72	80	0.000	0.000	0.000	0.000			NA	0.000		0.060
	**CTCF-11**	12.5		2%	19%	4%	700	8%	86%	56	90	0.018	0.111	0.000				0.000	0.000	0.009	0.105
	**CTCF-12**	12.5	3%	4%	1%	16%	1520	21%	78%	319	110	0.000	0.240	0.007				0.000	0.000	0.000	0.002
	**CTCF-13**	12.5	6%	21%	3%	11%	680	32%	68%	218	120	0.021	0.344					0.000	0.000	0.000	0.000
	Average		3%	8%	4%	9%															

Mice not included in group :No spleen weight /WBC count was available for the mice, so couldn't include in any group

**CTCF-5**	10		dead							230		1.672	0.917				0.029	0.200	0.490	
**CTCF-16**	10		dead														0.000	0.000	1.490	
**CTCF-17**	10	52%	dead														0.000	0.000	0.008	
