# Supplementary material for: Role of the CTCF binding site in Human T-Cell Leukemia Virus-1 pathogenesis
Source: PLoS Pathog. 2025 Jun 3;21(6):e1012293. doi: 10.1371/journal.ppat.1012293 (PMC12165413; doi:10.1371/journal.ppat.1012293)
Supplement: S4 Table — Columns include the number of individual cells within each clone for the ten most abundant clones in each sample; the V(D)J and CDR3 read for TRB and TRA for each clone; the T cell subtype of each clone (Treg, CD4, CD8, Double Positive); and the number of HTLV reads detected per clone. (PDF) [file ppat.1012293.s021.pdf]

Table S4. TCR V(D)J Sequencing and Subtyping. Columns include; the number of individual cells within each clone for the ten most abundant clones in each sample; the V(D)J and CDR3 read for TRB and TRA for each clone; the T cell subtype of each clone (Treg, CD4, CD8, Double Positive); and the number of HTLV reads detected per clone.

Treg
CD4
CD8
DP

	TRB V	TRB D	TRB J	TRB CDR3	TRA V	TRA D	TRA J	TRA CDR3	CD4+ (Count)	CD8+ (Count)	CD25+ (Count)	CD4+ (Percent)	CD8+ (Percent)	CD25+ (Percent)	HTLV Count
CTCF-7															
91	V11-2		J2-2	CASSYRGGGTGELFF	V36/DV7		J53	CAVKRWDSGGSNTKLTF	63	1	59	69	1	65	5
68	V7-2		J2-5	CASSLRTGQETQYF	V17		J52	CATPYGKLTF	39	61	17	57	90	25	2
43	V19		J2-7	CASSITQFYEQYF	V30		J52	CGSNAGGTSYGKLTF	19	1	22	44	2	51	1
42	V12-4		J1-1	CASSGGRILEAFF	V29/DV5		J34	CAASPLTWGNTDKLIF	1	42	2	2	100	5	0
39	V7-2		J2-5	CASSLRTGQETQYF					20	30	14	51	77	36	2
33	V5-6		J2-5	CASSLPWAWGVGETQYF	V38-2/DV8		J56	CAYRHRADTGANSKLTF	23	0	5	70	0	15	1
28	V28		J2-7	CASSPDRARQYF	V35		J47	CAGRRGGNKLVF	18	0	15	64	0	54	0
22	V5-1	D1	J2-3	CASSLWTGASLNHSSTDTQYF	V12-3		J21	CAMSLSYWNFNKFYF	3	22	0	14	100	0	0
18	V6-1		J1-5	CASNVIGARQPQHF	V38-2/DV8		J57	CAMNDSGGGADGLTF	0	17	1	0	94	6	1
16	V4-3	D2	J2-2	CASSQGGLAGGWTGELFF	V13-1		J8	CAASISFQKLVF	1	15	4	6	94	25	2
CTCF-8															
180	V29-1	D1	J1-1	CSVTGAMNTEAFF	V19		J52	CALSAYASYGKLTF	94	3	32	52	2	18	2
36	V4-2		J2-1	CASSQRSSYNEQFF	V17		J44	CATDAGTASKLTF	20	1	12	56	3	33	0
34	V12-5		J1-1	CASVGGWNTEAFF	V30		J52	CGAGGTSYGKLTF	6	22	0	18	65	0	0
25	V4-3	D1	J1-1	CASSQDIVGSAGTEAFF	V29/DV5		J6	CAASARSGGSYIPTF	4	19	3	16	76	12	1
20	V7-2		J2-7	CASSSDDRGRPYEQYF	V4		J21	CLVSLVYNFNKFYF	15	0	8	75	0	40	0
18	V7-9		J2-7	CASSFRDGYEQYF	V17		J56	CATGATGANSKLTF	10	0	4	56	0	22	0
17	V19		J1-2	CASSIVRVSNGYTF	V12-2 & V26-2		J10 & J40	CAVYLTGGGNKLTF & CILSADGTSGTYKYIF	13	0	1	76	0	6	0
17					V19		J52	CALSAYASYGKLTF	8	0	1	47	0	6	0
16	V10-2		J1-2	CASSDRGGGYTF	V13-1		J50	CAASIQTSYDKVIF	9	0	3	56	0	19	0
16	V12-5		J2-3	CASGEGQGSTDTQYF	V17		J23	CATGTYNQGGKLIF	10	1	3	63	6	19	2
CTCF-15															
84	V28		J2-7	CASSHRQSSYEQYF					51	0	25	61	0	30	0
61	V9		J2-7	CASSVAGGSYEQYF	V8-3		J42	CAVPYGGSQGNLIF	13	60	38	21	98	62	0
30	V7-3		J2-2	CASSFHGLAGLMNTGELFF	V26-2		J28	CIRIHRSGAGSYQLTF	15	0	6	50	0	20	0
27	V6-5		J1-6	CASSYDGGRPYNSPLHF	V8-1		J23	CAVNPLGGKLIF	12	0	10	44	0	37	0
26	V12-5 & V2	D1	J1-1 & J2-7	CASGLVEARTGEAEAFF & CASRGDRRYEQYF	V8-2		J26	CVVIYRNYGQNFVF	12	0	4	46	0	15	0
21	V5-5		J2-7	CASSGQGSYEQYF	V5		J40	CAEIPLSGTYKYIF	5	0	2	24	0	10	0
17	V7-8		J1-1	CASSLASRARTEAFF	V27 & V26-1		J49 & J40	CAGIPNTGNQFYF & CIVRALTTSGTYKYIF	0	7	0	0	41	0	0
16	V3-1		J2-5	CASSQSPGVQETQYF	V8-1		J8	CAVIARGFQKLVF	5	0	5	31	0	31	1
10	V5-6	D1	J1-2	CASQPGAGGYTF	V22		J21	CAVIYNFNKFYF	0	10	1	0	100	10	0
9	V28		J2-3	CASSFGLAGLTDTQYF	V14/DV4		J17	CAMREWAAGNKLTF	5	1	2	56	11	22	0
P12-10A															
42	V4-2		J2-6	CASSLQAGANVLTF	V35 & V39		J32 & J45	CAGLSGATNKLIF & CAVDITGGGADGLTF	29	0	13	69	0	31	1
25	V19		J1-2	CASSIPYGYTF	V21		J47	CAVSQYGNKLVF	22	3	14	88	12	56	0
21	V6-6	D1	J1-2	CASSWYRVPLYGYTF	V12-3		J49	CAIVTGNQFYF	1	21	1	5	100	5	0
16	V27		J2-2	CASSFPGGTGELFF	V25		J22	CAGPGSARQLTF	4	0	3	25	0	19	0
13	V4-1	D1	J1-1	CASSHTGTGDTEAFF	V38-1		J21	CAPSPYNFNNFYF	9	0	0	69	0	0	0
8	V6-5		J2-3	CASSRGARTDTQYF	V29/DV5		J48	CAASASDFGNEKLTF	0	6	3	0	75	38	0
7	V7-3		J2-2	CASSDGPNTGELFF	V9-2		J26	CALYNYGQNFVF	0	7	1	0	100	14	0
6	V6-6		J1-5	CASSEGARNQPQHF	V38-1		J40	CAFVSTSGTYKYIF	0	5	0	0	83	0	0
5	V28		J1-5	CASSLRQVLRNQPQHF	V39		J5	CAVSRYMDTGRRALTF	0	3	0	0	60	0	0
4	V18		J2-5	CASSPYSQETQYF	V8-4		J9	CAVSFYTGGFKTIF	0	4	0	0	100	0	0
P12-10B															
30	V6-5		J1-1	CASSFTPGPNTEAFF	V29/DV5		J44	CAASVFTGTASKLTF	20	2	22	67	7	73	0
27	V2		J1-1	CASSEGQVEAFF	V29/DV5 & V38-2/DV8		J43 & J53	CAASALGNNNDMRF & CAYSWGSNYKLTF	17	0	20	63	0	74	1
26	V20-1		J2-7	CSAHDRRYEQYF	V9-2		J57	CALARRGGSEKLVF	14	1	21	54	4	81	1
23	V30		J1-6	CAWNPGHFNSPLHF	V21		J13	CAVNSGGYQKVTF	8	0	12	35	0	52	1
23	V5-4	D1	J2-1	CASSLAYRTGYNEQFF	V35		J43 & J53	CAGRLPNNDMRF	17	0	12	74	0	52	1
22	V4-3		J2-1	CASSQTGMMGNEQFF	V19 & V8-2		J39 & J47	CALRVNNAGNMLTF & CVVSVGNKLVF	2	17	7	9	77	32	3
21	V-20		J2-7	CSAHDRRYEQYF					9	0	11	43	0	52	1
19	V5-4		J1-1	CASSLRALNTEAFF	V26-1		J58	CIVRVVETSGSRLTF	11	3	8	58	16	42	0
19	V20-1		J2-3	CSADGGAGGPTDTQYF	V26-1		J54	CIVTQGAQKLVF	15	1	11	79	5	58	0
19					V36/DV7		J54	CALRDLQGAQKLVF	9	0	11	47	0	58	1
P12-12															
34	V6-1		J1-1	CASSFRTAMNTEAFF	V30		J53	CGTVRGGSNYKLTF	31	1	28	91	3	82	0
34	V4-3		J2-3	CASSPRFTVRSTDTQYF	V8-4		J4	CAGMFSGGYNKLIF	4	31	17	12	91	50	1
24	V6-4		J1-2	CASSALPRTAIYGYTF	V35 & V51		J54 & J54	CAGFQGAQKLVF & CAVCDQGAQKLVF	11	0	1	46	0	4	0
19	V29-1		J2-7	CSVEVVGVGVSYEQYF	V8-1 & V1-2		J28 & J31	CAGPGAGSYQLTF & CAVNNARLMF	0	17	0	0	89	0	0
18	V5-6		J1-3	CASSFGGLVTGNTIYF	V21		J28 & J31	CAVRQDGAGSYQLTF	0	17	10	0	94	56	0
14	V6-5		J2-1	CASSTGGEQFF	V29/DV5		J22	CAALLSGSARQLTF	7	13	11	50	93	79	0
11	V7-9		J2-7	CASSALIQDEQYF	V17		J31	CATGTNARLMF	2	11	5	18	100	45	1
9	V10-3	D1	J2-5	CAIRQGGEGTQYF	V41		J31	CAVEGNARLMF	6	0	6	67	0	67	0
9	V15		J1-5	CATSSGDRLNQPQHF	V26-1		J32	CIVRPPYGGATNKLIF	0	9	0	0	100	0	1
9	V11-1		J2-7	CASSFDRAGGPYEQYF	V17		J33	CATGPGYYYQLIW	5	8	5	56	89	56	1
P12-14															
191	V28		J2-4	CASSLGWYLAKNIQYF	V30 & V26-1		J49 & J21	CGTGYRDTGNQFYF & CIVRVPYNFNKFYF	71	179	42	37	94	22	4
96	V7-8		J2-4	CASSLAQGISKNIQYF	V14/DV4		J13	CAMREGWGYQKVTF	3	90	10	3	94	10	3
45	V7-2		J1-1	CASSLDRMAAEAFF	V17		J37	CATGLGSNTGKLIF	3	41	12	7	91	27	1
44	V7-9		J1-4	CASSLLRRGEKLFF	V12-3		J57	CAMTAKRGGSEKLVF	36	43	10	82	98	23	1
32	V6-1	D1	J1-1	CASRGQPGQGHQAFF					14	0	15	44	0	47	1
30	V7-9		J2-5	CASSRQGSQETQYF	V30		J53	CGTERGGSNYKLTF	23	3	23	77	10	77	2
24	V28		J2-4	CASSLGWYLAKNIQYF					5	21	4	21	88	17	2
23	V27		J1-5	CASSSEARQPPHF	V13-1		J13	CAAFRRGYQKCTF	0	22	3	0	96	13	1
21	V4-1	D1	J1-2	CASSQDWAGDYGYTF	V13-2		J58	CAENGETSGSRLTF	14	1	11	67	5	52	0
20	V2		J1-4	CASSQQAYFNEKLFF	V21		J33	CAVKGSNYQLIW	14	1	13	70	5	65	1

T Cell Subtype (>20% highlighted)
