# Supplementary material for: Role of the CTCF binding site in Human T-Cell Leukemia Virus-1 pathogenesis
Source: PLoS Pathog. 2025 Jun 3;21(6):e1012293. doi: 10.1371/journal.ppat.1012293 (PMC12165413; doi:10.1371/journal.ppat.1012293)
Supplement: Attachment — Submitted filename: Response to Critiques.26MAR2025.docx [file ppat.1012293.s023.docx]

Dear Lee,

Thank you very much for submitting your manuscript "Role of the CTCF Binding Site in Human T-Cell Leukemia Virus-1 Pathogenesis" for consideration at PLOS Pathogens. As with all papers reviewed by the journal, your manuscript was reviewed by members of the editorial board and by several independent reviewers. In light of the reviews (below this email), we would like to invite the resubmission of a significantly-revised version that takes into account the reviewers' comments.

Thank you for your patience in awaiting feedback on your manuscript. We have now received comments from three reviewers. While all three found your results of interest, all also made a considerable number of comments and suggestions regarding the phrasing of the paper and, more substantially, the interpretation of the experimental results and the extent of the claims made.

We cannot make any decision about publication until we have seen the revised manuscript and your response to the reviewers' comments. Your revised manuscript is also likely to be sent to reviewers for further evaluation.

When you are ready to resubmit, please upload the following:

[1] A letter containing a detailed list of your responses to the review comments and a description of the changes you have made in the manuscript. Please note while forming your response, if your article is accepted, you may have the opportunity to make the peer review history publicly available. The record will include editor decision letters (with reviews) and your responses to reviewer comments. If eligible, we will contact you to opt in or out.

[2] Two versions of the revised manuscript: one with either highlights or tracked changes denoting where the text has been changed; the other a clean version (uploaded as the manuscript file).

Important additional instructions are given below your reviewer comments.

Please prepare and submit your revised manuscript within 60 days. If you anticipate any delay, please let us know the expected resubmission date by replying to this email. Please note that revised manuscripts received after the 60-day due date may require evaluation and peer review similar to newly submitted manuscripts.

Thank you again for your submission. We hope that our editorial process has been constructive so far, and we welcome your feedback at any time. Please don't hesitate to contact us if you have any questions or comments.

With best wishes,

Charles

Charles R M Bangham, ScD FRS
Academic Editor
PLOS Pathogens

Richard Koup
Section Editor
PLOS Pathogens

Michael Malim
Editor-in-Chief
PLOS Pathogens
orcid.org/0000-0002-7699-2064

***********************

Thank you for your patience in awaiting feedback on your paper. We have now received comments from three reviewers. While all three found your results of interest, all also made a considerable number of comments and suggestions regarding the phrasing of the paper and, more substantially, the interpretation of the experimental results and the extent of the claims made.


Reviewer's Responses to Questions

Authors’ Response to Reviewer’s Comments – All page and line numbers refer to the tracked version of the manuscript.

**Part I - Summary**
Please use this section to discuss strengths/weaknesses of study, novelty/significance, general execution and scholarship.

Reviewer #1: In the present study, Joseph et al. investigate the in vivo role of the HTLV-1-CTCF binding site (vCTCF-BS), using 2 humanized mouse models.
Infection was initiated with 729B cells transfected with: i) HTLV-1-WT, ii) HTLV-1-CTCF, which contains a provirus with a mutated vCTCF-BS, and a stop codon, which deletes the last 23 amino acids of p12, and iii) HTLV-1-p12stop, which contains the intact vCTCF-BS, but retains the same stop codon in p12.
Based on their observations, the Authors suggest a key role of the CTCF binding site in HTLV-1 replication and pathogenicity in vivo.

Reviewer #2: In Joseph et al, the authors assess the role of the HTLV-1 CTCF binding site in viral replication and pathogenesis in vivo using a humanized mouse model. Mutation of the vCTCF-BS delayed virus spread and delayed or abrogated lymphoproliferative disease in HTLV-1 infected Hu-mice. The data further suggest that loss of the vCTCF-BS results in significant effects on host gene expression and expansion of human T cell populations in vivo, including suppression of activity of CD8+ T-cells, and that the vCTCF-BS has dynamic regulation of HTLV-1 gene expression.

Interestingly, previous in vitro studies carried out by the same group revealed that mutation of the vCTCF-BS did not disrupt the kinetics and levels of virus gene expression. Moreover, no effects on minus strand gene expression were seen in vitro.

Response: Lines 344-349 in the Discussion point out that the previous work was performed in immunocompetent rabbits, whereas the current work was performed in humanized immunodeficient mice.

This is an interesting study for the field. It exploits humanized mouse models to unravel the role of a unique CTCF motif present in the HTLV-1 provirus on the regulation of both the host genome in which it integrates and the viral genome in vivo. A weakness of the study is that the impact of the viral CTCF on Tax expression was assessed in an in vitro model, while there was no evidence for this effect in Hu-mice in vivo.

Response: Lines 468-471 in the Discussion point out this limitation, but it is beyond the current work and technology to confirm *in vitro* results shown in Fig 6 with *in vivo* approaches.

Reviewer #3: Joseph et al. present an investigation into the role of the viral CTCF binding site in a mutated virus that encodes a truncated p12 protein, in a humanised mouse model of infection and pathogenesis. Authors observe a number of markers that are consistent with reduced pathogenicity upon mutation of the CTCF binding site. The manuscript is very clearly written, and easy to follow. However, there are several confounding factors that make it difficult to interpret the data presented.

Response: As described below, we simplified the analysis by separating the data concerning the effects of p12 truncation from those related to analysis of the vCTCF-BS, and moving all data with HTLV-1-WT to Supplementary Figures, and limiting the key Figures in the paper to a comparison of HTLV-1-p12stop with HTLV-1-CTCF infections.

**Part II – Major Issues: Key Experiments Required for Acceptance**
Please use this section to detail the key new experiments or modifications of existing experiments that should be absolutely required to validate study conclusions.

Generally, there should be no more than 3 such required experiments or major modifications for a "Major Revision" recommendation. If more than 3 experiments are necessary to validate the study conclusions, then you are encouraged to recommend "Reject".

Reviewer #1: In general, the manuscript is written in a rather convoluted and, at points, confusing manner.

Response: Confounding effects that led to confusion are addressed above in response to Summary Comment of Reviewer #3

Although addressing the relevant question of the importance of the CTCF in HTLV-1 infection in vivo, the study is weakened by several drawbacks.
• The p12 stop codon and mutations introduced in the CTCF binding site also change amino acids in the viral regulatory protein p30 (80V to D; 83R to L) and truncate p8. The introduced mutations may also change HBZ non-coding RNA, which is known to be critical for T-cell immortalization. All these consideration make it difficult to pinpoint any of the observed differences solely to the function of the CTCF binding site. Consistent with this concern, in many of the analyses the p12stop virus seems to differ markedly from WT (e.g. Fig. 2).

• Many of the differences are observed between HTLV-1-CTCF and p12 stop, but not WT (see Figs. 1 and 2).

Response: Our previous 2021 study showed no effect on known p12 or HBZ functions with these changes. However, to avoid confusion, we focused the analysis on comparison of HTLV-1-p12stop and HTLV-1-CTCF infected animals (see response to Summary critiques of Reviewer #3 above).

• In Fig. 2C the Bone Marrow plot should show a red bar for p12stop and a green bar for HTLV-1-CTCF. Was bone marrow analyzed in the WT virus group?

Response: The color of the bars in the figure were corrected. Bone marrow was analyzed in the HTLV-1-WT infected animals and is shown in the revised Fig S1.

• The 2 humanized models yield somewhat different results.

Response: The humanized models are different models, as described on lines 89-92 in the Introduction of the revised manuscript. However, in both models HTLV-1-CTCF mice showed lower proviral loads (revised Fig 1 and Fig S4) and attenuated or no disease as compared to HTLV-1-p12stop and HTLV-1-WT mice (revised Fig S1).

• In the CD133 Hu model the experimental groups’ numerosity is not well matched (WT N=7, CTCF N=6, p12stop N=3). In the CD34 Hu model, the experimental groups’ numerosity is not indicated.

Response: In the CD34 Hu model, numerosity is now indicated in each figure legend, and provided in the new Supplemental Table S1

• The scRNAseq image is of extremely poor quality, making it hard to interpret the results. In which cell subset did the Authors see HTLV-1 expression? In which cell types does HTLV-1 persist?

Response: A revised figure for HTLV-1 expressing cells is provided in Fig 5B and noted in the Results on the line 270 that the majority of HTLV-1 expressing cells are CD4+ cells, as can be seen by comparing revised Fig 5A to 5B.

• The Authors suggest that lack of CTCF results in delayed onset/smoldering disease. This statement needs to be validated by testing the expression levels (or function) of CTCF in samples form HC vs. smoldering or acute ATL.

Response: Lines 428-431 in the Discussion point out that “Although mutation of the vCTCF-BS results in an asymptomatic infection or smoldering lymphoproliferative disease in humanized mice rather than an acute leukemic or lymphomatous form of disease, it is premature to suggest that this specific genetic element alone determines the disease phenotype in infected humans.”

Reviewer #2: (No Response)

Reviewer #3: Major Concerns
● Authors state that the p12-stop mutant is comparable to WT virus in cellular assays. However, throughout this manuscript, there are many instances where the p12-stop mutant behaves differently to the WT virus, seemingly increasing the pathogenicity of HTLV-1. In line 145, authors attribute differences between wt and p12-stop to sampling errors, however a consistent trend seen throughout the manuscript suggests truncating p12 might indeed alter pathogenicity in HuMice, which is confounding. Other studies (PMID: 26929370) mutate the CTCF binding site without truncating p12. This mutant should also be included in the study, to control for both substitutions (as was a concern in previous work), and the truncation.

Response: Addressed above in response to Summary critique of Reviewer #3. The mutant examined by Miura et al. introduced 4 mutations in the p12 gene, and thus, it would not clarify the role of the vCTCF-BS vs p12.

● Line 172: While it is interesting that vCTCF BS mutant HuMice can be stratified into two groups that correlates with PVL, it is important to present this data much more comprehensively. In Fig. 4F, assignment of each mouse into group 1 or 2 needs to be explicitly marked, and in all panels in Fig 4. Data from WT and P12 controls needs to be included in all the main figure panels, especially as authors state that group 1 mice behave similarly to controls (line 176).

Response: Addressed above in response to Summary critique of Reviewer #3 as well as by providing a new Supplementary Table 1 with data for all mice.

● It would be interesting to know whether mice that stratify into two groups behave differently at early time points. Is the PVL consistently higher throughout the study? There are some datapoints in the vCTCF BS mutant mice that seem more comparable to controls, are these the group 1 mice? Given the variability seen in this condition, readers would benefit from a supplementary table, summarising each mouse used throughout the study, including information such as levels of human CD45+ cells, PVL at each time point, disease status, etc.

Response: New Supplementary Tables 1-3 are provided with data for all mice.

● Proceeding to perform scRNA-seq on only group 2 vCTCF BS mutants biases for those with a low PVL and no markers of disease progression, and therefore analysis is confounded, and changes in gene expression or cell populations cannot be attributed to perturbation of CTCF binding alone. RNA-seq should be performed on group 1 samples as well, and should be matched with controls for equivalent PVL from both WT and p12-stop groups, to ascertain the effect of CTCF binding.

Response: Revised Supplementary Figure S8 provides results with all 7 scRNAseq analyses. Lines 219-223 in Results also states the following: “Single cell RNAseq analysis was performed on HTLV-1-CTCF-A mice with the highest proviral loads (Figure 5). Sc RNAseq analysis performed on a HTLV-1-CTCF-B mouse could not be analyzed due to lack of adequate numbers of human cells. Levels of human cells in splenocytes of HTLV-1-CTCF2 mice were 4.5-fold lower than those in HTLV-1-CTCF1 mice (Fig 3E).”

● Representative data in Fig S4. is really informative. It looks as though there may be some heterogeneity in HTLV-1 transcript expression, as has been observed before. Is this the same for HTLV-1 expressing cells? The tSNE presented in Fig 5B should be presented for each sample as a supplementary figure and expanded to show the different transcripts (single spliced, double spliced, spliced HBZ) as was done in S10 for ALOX5AP expression, and Fig S4 A and B should be expanded to show genome browser tracks for all 7 samples.

Response: Revised Fig S8 shows the transcripts in each of the 7 animals examined by scRNAseq. Fig S15 shows tSNE plots for each of the 7 animals.

● Although the in vitro data presented in Fig 6. is really compelling, the model in Fig 7. is difficult to address while concerns around RNA-seq, and thus functional effects of abrogating viral CTCF binding in vivo, have not been resolved

Response: As stated above in response to Summary critique of Reviewer #2:

Lines 468-471 in the Discussion point out this limitation, but it is beyond the current work and technology to confirm the *in vitro* results of Fig 6 results with *in vivo* approaches.

**Part III – Minor Issues: Editorial and Data Presentation Modifications**
Please use this section for editorial suggestions as well as relatively minor modifications of existing data that would enhance clarity.

Reviewer #1: n/a

Reviewer #2: Main comments

Introduction
Tax is expressed intermittently in a small proportion of ATLL cells at any given time [4, 5].
Is this consistently observed in any ATLL case? A proportion of ATLLs carry a tax-deficient provirus, either a mutation in the tax ORF that abolishes the transactivation or oncogenic potential of HTLV-1, or a 5’LTR-deficient provirus that has lost the capacity to express Tax. Did the authors examine nucleotide sequences of such proviruses, and is the CTCF consensus sequence conserved in these proviruses (> 30% of ATLLs).

Response: We edited lines 74-76 of the Introduction to say:

“There is a single viral CTCF-binding site (vCTCF-BS) in HTLV-1, which is conserved in almost all HTLV-1 strains and other delta-retroviruses [9-11].”

The authors use two distinct methods for humanizing NBSGW mice, intrahepatic injection of human cord blood CD34+ cells, and intratibial injection of CD133 progenitor cells. They describe the CD34+ model at the end of the introduction and refer to Fig 1A. This figure illustrates both the CD34- and CD133-driven models, CD133 being shown on top. It is confusing for the reader to have this figure in the introduction, while CD133 has not yet been described. We suggest to move the figure from the introduction section to Results, with CD34 on top, and CD133 at the bottom (invert).

Response: The change proposed for Fig 1A was made.

The reason for using the CD133 model is not clear. The results (line 122-134), although suggesting decreased pathogenicity of the CTCF infected mice, are obviously different, however the numbers insufficient for statistical analysis.

Response: The rationale for use of CD133 animals is added to the Introduction on lines 89-92:

Two different humanized mouse models were used that more closely simulate the effects seen in lymphomatous (infection of humanized mice with intrahepatic injection of newborn mice with CD34+ HSCs) and acute leukemic (infection of mice humanized with CD133+ HSCs injected in the tibia) of ATLL in patients.

Fig S3: p12stop infected mice (claimed to be the better control) do also have lower proviral loads. The p-values are estimated for CTCF versus wt-infected animals (3B) not CTCF versus p12stop.

Response: p values are provided for all comparisons in revised Fig S1, 2. For revised Fig S1, S2, S3, S4, and S5.

Monitoring disease: how is disease measured during the course of infection before death, are signs of disease only assessed by body weight loss (dropped by 20% or more of their initial body mass), or is this also based on cell counts in the blood? Do the mice die from the disease in some cases (line 166: succumbed from infection) or are they systematically euthanized according to the weight loss criteria indicated above? How do you distinguish between a benign lymphoproliferation and leukemia/lymphoma (malignant disease)? Is it correct that mice do never develop “leukemia” (acute ATLL in human) but only “lymphoma” (lymphoma-subtype ATLL in human)?

Response: Parameters used in monitoring animals are listed in Results on lines 120-122 and Methods on lines 530-531

Also, we added in the Results section that experiments were terminated at 12.5 wks and all animals alive at that point underwent necropsy. This time point was selected as a time point which is 3 standard deviations beyond the mean survival of p12stop animals, as indicted on lines 125-127 in the Results.

Line 99: Mice were then inoculated intraperitoneally with …. An equal number of mice were also infected with HTLV-1 with the same mutation found in the HTLV-1-p12stop virus, as well as an additional mutation that abrogates the vCTCF-BS (HTLV-1-CTCF). Eight litters of mice were used to obtain a sufficient number of mice for statistically valid results, with similar numbers, sexes, and levels of CD34+ cells in mice within each litter allocated for infection with HTLV-1-WT, HTLV-1-p12stop, or HTLV-1-CTCF.

Response: Revised Tables S1 and S2 provided all the requested information for each and every animal

Provide specific numbers for the CD34+ experiment. Numbers are given for the CD133 experiment (Infection with HTLV-1-CTCF (n=6) resulted in delayed onset of lymphoproliferative disease compared to mice infected with HTLV-1-WT (n=7) or HTLV-1-p12stop (n=3) (Figure S1A). For the main model, CD34+, how many animals in each group? Among each group, how many were assessed by scRNA-seq? How many are shown in the figures and how were they selected. Show data of remaining animals in Supplemental material.

Response: Numbers of animals in each experiments are added to figure legends and new Supplementary Tables 1-3 provide data of all animals.

Example: How did the authors select the animals (7) examined by scRNA-seq? How many did develop lymphoproliferative disease in each group? In Fig S3, sequencing reads of 4 mice are illustrated in IGV while 7 mice have been sequenced. Do the three other samples have similar transcriptional patterns (provide the number of reads observed for each spicing type if not showing the reads in the figure)?

Response: We added to the Results section that

“Single cell RNAseq analysis was performed on HTLV-1-CTCF-A mice with the highest proviral loads (Figure S5). Scl RNAseq analysis performed on a HTLV-1-CTCF-B mouse was insufficient due to lack of adequate numbers of human cells. Levels of human cells in splenocytes of HTLV-1-CTCF-B mice were 4.5-fold lower than those in HTLV-1-CTCF1 mice (Fig 3E).

Role in HTLV-1 pathogenesis:
The stratification in CTCF-1: n=8 and CTCF-2: n=7
Fig 4F illustrates the inverse correlation between PVL at 5 weeks and survival. 14/15 mice are shown, how did the 15th mouse behave? For each of the CTCF mice shown in Fig 4F, indicate to which category it belongs, CTCF-1 or CTCF-2.

Response: This is indicated in the revised Fig 3F as well as providing data on individual mice in new Supplementary Tables S1 and S2

scRNA-seq transcriptional profiles: Fig 5B indicates color labels for CD4+, CD8+, CD25+ and TCR+ cells (clustering), it also indicates “T-cells” (blue label in the legend). Clarify how colors are used, and do they overlap? i.e. CD4+ cells are also TCR+ etc … Regarding viral reads in scRNA-seq data, the data were described earlier in the manuscript (line 155 and further). It feels confusing for the reader to have this described at two distinct places in the paper, especially that the first description does not mention the CTCF-2 distinction and that only these mice were used for scRNA-seq analysis.

Transcriptional profiles: T cell clonality was high for all samples as revealed by Gini coefficient and Shanon Diversity index. This quantifies the clonal distribution in terms of TCR and does not discriminate between infected and uninfected T-cell clones. Providing a measure of HTLV-1 integration site distribution by assessing viral clonality using DNA of the same tissues will provide an estimate of the nature and relative integration site abundances even in low PVL animals.

RNA-seq data and Supplementary table: while the TCR sequences are informative, HTLV-1 reads per clone are not a good proxy for the presence of the virus since many cells carrying the virus do not express viral genes, or do express these genes at a low level not detectable via scRNA-seq. Examining HTLV-1 clonality in these cells is technically possible and will define the viral IS landscape. Data can then be combined with TCR clonality (RNA, table) and the number of infected cells extrapolated via PVL values to provide a global T-cell/HTLV-1 clonality picture in CTCF versus p12stop animals.

Response: We added the following on lines 276-277 in the Results

“Integration site clonality assay was performed on two HTLV-1-p12stop and two HTLV-1-CTCF-A mice for which sufficient DNA was available.” There was not adequate DNA from the other three animals on which scRNAseq assays were performed for the clonality assay.

Role of CTCF on Tax expression
Line 365: … support a potentially new discovery that CTCF regulates TAX in vivo.
There is no evidence for this statement from this study in Hu-mice. scRNA-seq data did not reveal significant differences in Tax expression between CTCF and p12stop infected animals. This assumption is based on in vitro work, which, although elegant and convincing by itself in the Jet model system, does not confirm this effect in vivo. The authors may think about a design to better exploit scRNA-seq, i.e. perform scRNA-seq of the splenocytes (human lymphocytes) collected from the same Hu-mice ex vivo and after in vitro culture, or propose an in vivo experiment to assess this assumption.

Response: As stated above, we added on lines 468-471 in the Discussion:

“A limitation of the current work is that the effects of CTCF on Tax expression were performed in tissue culture models and remain to be confirmed *in vivo*. Such studies require development of more highly sensitive assays of virus expression and replication over the course of the first few days of infection *in vivo*.”

In the abstract: Overall, these findings indicate that the vCTCF-BS regulates Tax expression, proviral load, and HTLV pathogenicity in vivo. The effect on Tax expression in vivo was not demonstrated, therefore, this statement should be rephrased.

Response: Fig S8A showed no significant differences in tax expression at necropsy.

scRNAseq:
Information in the scRNA-seq paragraph of the Methods section is scarce and should be further explained. What was the viability of the cells used for scRNA-seq and how could the authors differentiate between viable mouse cells and viable human lymphocytes? Custom analysis: what does this mean, the type of analysis, how and why it was performed should be clarified.

Response: More detailed methods are provided on lines 581-592.

Figure 5: B: TSNE plots of a representative data set: clarify. A single animal? How was this animal selected? Line 201: Characteristics of predominant T cell clones, including CD4+, CD8+, Treg, and double CD4+CD8+ clones, varied in each sample. The six remaining datasets should be reported in Supplemental material.

Response: tSNE plots for all 7 animals provided in revised Fig S15

Minor comment:

Line 218: “mice infected HTLV-1-CTCF-2 and HTLV-1-p12stop” -> mice infected WITH HTLV-1-CTCF-2 and HTLV-1-p12stop

Response: Corrected

Reviewer #3: Minor Issues
● If PVL was undetectable in many mice, particularly those harbouring CTCF mutant virus, how was infection confirmed?

Response: Confirmation of infection is added in the Methods on lines 535-536:

“

● Authors should state the rationale for using the two complementary methods for generating mice

Response: Stated in Introduction on lines 89-92:

Two different humanized mouse models were used that more closely simulate the effects seen in lymphomatous (infection of humanized mice with intrahepatic injection of newborn mice with CD34+ HSCs) and acute leukemic (infection of mice humanized with CD133+ HSCs injected in the tibia) of ATLL in patients.

Ref

● Line 60: typo - pr instead of pro

Response: Corrected

● Lines 75-77: CTCF’s role is oversimplified to the point where it is inaccurate. It primarily functions as an insulator protein, which acts by restraining cohesion mediated loop extrusion, and is associated with both transcriptional activation and repression of cellular genes.

Response: Corrected to indicate that CTCF is a chromatin insulator

● Line 156: “Interestingly, unlike in infected cells in tissue culture [14], hbz transcripts were the most abundant viral mRNAs in infected splenocytes in vivo” and this is also unlike in individuals with HTLV-1 (PMID: 29062917, PMC4847349) however, it’s worth highlighting that individuals with ATL have higher levels of hbz transcripts, which is consistent with lymphoproliferative disease development seen in the Hu-mice

Response: Highlighted in revised Discussion on lines 229-232

“Interestingly, unlike in infected cells in tissue culture [17], *hbz* transcripts were as abundant as plus strand viral transcripts in infected splenocytes *in vivo* (Figure S8A) for both HTLV-1-CTCF (n=3) and HTLV-1-p12stop infected Hu-mice (n=4).”

● More detail is needed for Fig S9A. and S11B legend. Volcano plots are showing log2FC between what conditions?

Response: More detail is provided for volcano plots in revised Fig S14 and S16

● Legend for Fig S4: causes confusion to say “BAM files of unmapped reads from a subset of 4 samples on IGV”, convention would be to say reads mapping to the HTLV-1 proviral genome

Response: Changed as recommendced

PLOS authors have the option to publish the peer review history of their article ([what does this mean?](https://nam10.safelinks.protection.outlook.com/?url=https%3A%2F%2Fjournals.plos.org%2Fplospathogens%2Fs%2Feditorial-and-peer-review-process%23loc-peer-review-history&data=05%7C02%7Clratner%40wustl.edu%7C448d1edd4f514b9b977d08dcb09b275a%7C4ccca3b571cd4e6d974b4d9beb96c6d6%7C0%7C0%7C638579427639203583%7CUnknown%7CTWFpbGZsb3d8eyJWIjoiMC4wLjAwMDAiLCJQIjoiV2luMzIiLCJBTiI6Ik1haWwiLCJXVCI6Mn0%3D%7C0%7C%7C%7C&sdata=qE3VFq4XaMl%2FYieM80NRz%2Fz4FxVa1RXKUwQkqF0kxFA%3D&reserved=0)). If published, this will include your full peer review and any attached files.

If you choose “no”, your identity will remain anonymous but your review may still be made public.

**Do you want your identity to be public for this peer review?** For information about this choice, including consent withdrawal, please see our [Privacy Policy](https://nam10.safelinks.protection.outlook.com/?url=https%3A%2F%2Fwww.plos.org%2Fprivacy-policy&data=05%7C02%7Clratner%40wustl.edu%7C448d1edd4f514b9b977d08dcb09b275a%7C4ccca3b571cd4e6d974b4d9beb96c6d6%7C0%7C0%7C638579427639226224%7CUnknown%7CTWFpbGZsb3d8eyJWIjoiMC4wLjAwMDAiLCJQIjoiV2luMzIiLCJBTiI6Ik1haWwiLCJXVCI6Mn0%3D%7C0%7C%7C%7C&sdata=uEcGrJ5ZGmq%2B2li7AWJRlEsEXPudYH7PeeQpYXXg0AI%3D&reserved=0).

Reviewer #1: No

Reviewer #2: No

Reviewer #3: No

Figure Files:

While revising your submission, please upload your figure files to the Preflight Analysis and Conversion Engine (PACE) digital diagnostic tool, [https://pacev2.apexcovantage.com](https://nam10.safelinks.protection.outlook.com/?url=https%3A%2F%2Fpacev2.apexcovantage.com%2F&data=05%7C02%7Clratner%40wustl.edu%7C448d1edd4f514b9b977d08dcb09b275a%7C4ccca3b571cd4e6d974b4d9beb96c6d6%7C0%7C0%7C638579427639238540%7CUnknown%7CTWFpbGZsb3d8eyJWIjoiMC4wLjAwMDAiLCJQIjoiV2luMzIiLCJBTiI6Ik1haWwiLCJXVCI6Mn0%3D%7C0%7C%7C%7C&sdata=cVKCgM6hZIk9EpcRHD0t9qCmJV%2BkjodgSTjgDTTs0zU%3D&reserved=0). PACE helps ensure that figures meet PLOS requirements. To use PACE, you must first register as a user. Then, login and navigate to the UPLOAD tab, where you will find detailed instructions on how to use the tool. If you encounter any issues or have any questions when using PACE, please email us at [figures@plos.org](mailto:figures@plos.org).

*Insure quality of all figures*

Data Requirements:

Please note that, as a condition of publication, PLOS' data policy requires that you make available all data used to draw the conclusions outlined in your manuscript. Data must be deposited in an appropriate repository, included within the body of the manuscript, or uploaded as supporting information. This includes all numerical values that were used to generate graphs, histograms etc.. For an example see here on PLOS Biology: <http://www.plosbiology.org/article/info%3Adoi%2F10.1371%2Fjournal.pbio.1001908#s5>.

*Submit genomic data*

Reproducibility:

To enhance the reproducibility of your results, we recommend that you deposit your laboratory protocols in protocols.io, where a protocol can be assigned its own identifier (DOI) such that it can be cited independently in the future. Additionally, PLOS ONE offers an option to publish peer-reviewed clinical study protocols. Read more information on sharing protocols at [https://plos.org/protocols?utm_medium=editorial-email&utm_source=authorletters&utm_campaign=protocols](https://nam10.safelinks.protection.outlook.com/?url=https%3A%2F%2Fplos.org%2Fprotocols%3Futm_medium%3Deditorial-email%26utm_source%3Dauthorletters%26utm_campaign%3Dprotocols&data=05%7C02%7Clratner%40wustl.edu%7C448d1edd4f514b9b977d08dcb09b275a%7C4ccca3b571cd4e6d974b4d9beb96c6d6%7C0%7C0%7C638579427639245046%7CUnknown%7CTWFpbGZsb3d8eyJWIjoiMC4wLjAwMDAiLCJQIjoiV2luMzIiLCJBTiI6Ik1haWwiLCJXVCI6Mn0%3D%7C0%7C%7C%7C&sdata=x4zLh5FUhpgf%2BHThtxVgIhNu6bFZSR8Gw%2FgqtEVUqXU%3D&reserved=0)

*Deposit protocols*
